# Effects of Specular Highlights on Perceived Surface Convexity

**DOI:** 10.1371/journal.pcbi.1003576

**Published:** 2014-05-08

**Authors:** Wendy J. Adams, James H. Elder

**Affiliations:** 1 Psychology, University of Southampton, Southampton, United Kingdom; 2 Centre for Vision Research, York University, Toronto, Canada; New York University, United States of America

## Abstract

Shading is known to produce vivid perceptions of depth. However, the influence of specular highlights on perceived shape is unclear: some studies have shown that highlights improve quantitative shape perception while others have shown no effect. Here we ask how specular highlights combine with Lambertian shading cues to determine perceived surface curvature, and to what degree this is based upon a coherent model of the scene geometry. Observers viewed ambiguous convex/concave shaded surfaces, with or without highlights. We show that the presence/absence of specular highlights has an effect on qualitative shape, their presence biasing perception toward convex interpretations of ambiguous shaded objects. We also find that the alignment of a highlight with the Lambertian shading modulates its effect on perceived shape; misaligned highlights are less likely to be perceived as specularities, and thus have less effect on shape perception. Increasing the depth of the surface or the slant of the illuminant also modulated the effect of the highlight, increasing the bias toward convexity. The effect of highlights on perceived shape can be understood probabilistically in terms of scene geometry: for deeper objects and/or highly slanted illuminants, highlights will occur on convex but not concave surfaces, due to occlusion of the illuminant. Given uncertainty about the exact object depth and illuminant direction, the presence of a highlight increases the probability that the surface is convex.

## Author Summary

A primary goal of the human visual system is to reconstruct the three-dimensional structure of the environment from two-dimensional retinal images. This process is under-determined: an infinite number of combinations of shape, material properties and illumination conditions could give rise to any single image. Rather than determining the true three-dimensional scene in a deductive manner, the visual system must make its ‘best guess’ based on the image, probabilistic models of image formation, and the stored probability of various scene configurations. For example, the visual system appears to assume that convex surfaces are more common than concave ones, biasing perception toward convex surfaces when the image is ambiguous. Here we identify a new probabilistic cue for surface shape: a shape with a visible specular highlight is more likely to be convex than one without. Highlights occur when light is reflected in a mirror-like way from glossy surfaces such as polished marble or metal. Due to the geometry of reflection, however, highlights are more likely to be occluded on concave objects. We show that the human visual system makes use of this constraint: shape perception is biased toward convex surfaces when highlights are apparent.

## Introduction

Shading can produce striking impressions of 3D shape. However, recovering shape from shading is far from straightforward; luminance variations in the image are determined not only by the object's shape but also by its reflectance and the illumination conditions. To estimate shape from shading, the visual system biases judgements toward more common scenes, for example, light sources that are roughly overhead (e.g. [1], [2]) and surfaces with homogenous reflectance, at least in the absence of hue variation [3]. Here we explore an additional regularity that the visual system appears to exploit in estimating surface shape: that specular highlights suggest convex, rather than concave curvature. We test this proposal psychophysically and show why, given certain assumptions, this bias is rational: it reflects a higher likelihood of observing a specular reflection from a convex object.

**Figure 1:**
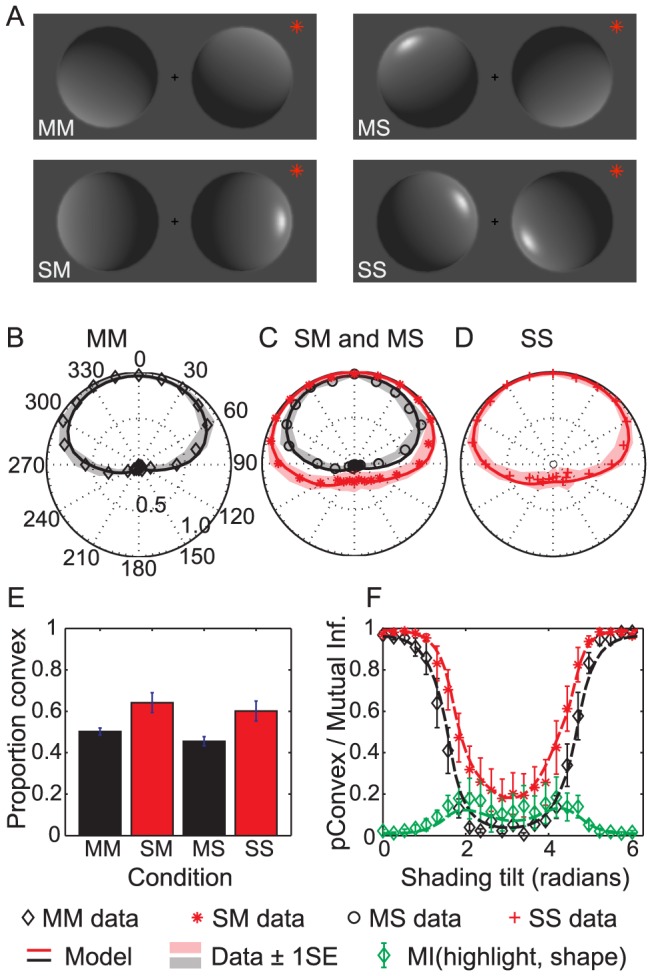
Experiment 1. (a) Example stimuli from the four conditions of Experiment 1. The star indicates which object is the target to be judged. The two-letter label denotes whether the judged object and the non-judged distractor were matte (M) or shiny (S), respectively. (b–d) Proportion of objects perceived as convex, as a function of shading direction (in deg), averaged across observers. The solid lines indicate the fit of the model to the averaged data. Shaded region indicates 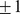 standard error (SEM) from the mean. (e) Proportion of convex responses for the four conditions, across observers. Error bars indicate ±1SEM across observers. (f) Perceived shape in the SM (red) and MM (black) conditions, and the corresponding mutual information (green diamonds) between the highlight and the perceived shape.

It is well known that a specular highlight can change the perception of surface material, making a matte object look glossy (Figure 1a). However, the effect of specular highlights on *shape* perception has received little attention. Specular highlights do carry shape information, tending to ‘cling’ to regions of high curvature [4]–[6], and observers can use the structure of specular highlights alone (e.g. on perfectly mirrored surfaces) to estimate curvature magnitude [7]. Several studies have compared monocular shape perception across matte and specular surfaces to assess the role of specular highlights in quantitative shape estimation. Whilst some studies found that specular highlights increased perceived depth [8]–[10] or improved shape discrimination [11], others have found no effect of surface specularity on shape judgements [12]–[14]. Ho, Landy and Maloney [15] found that the glossiness and bumpiness of a surface are somewhat confusable, even under binocular viewing: increasing surface depth increases perceived glossiness and vice versa.

When a glossy object is rotated, specular highlights glide across the object's surface, rather than being fixed to it like texture. This motion provides information that observers exploit to judge both gloss [16], [17] and shape [11], [18], [19]. Similarly, under binocular viewing, the disparity of specular highlights holds information not only about the magnitude of surface curvature but also its sign; for simple convex objects, specular highlights are stereoscopically behind the surface, for concave they are generally in front. The visual system appears to use this information in judgments of glossiness and, to a limited extent, shape [11], [20]–[24].

Note that this cue to surface convexity depends upon the binocular disparity of reflections. For distant surfaces, this disparity signal will be weak, and thus no bias is predicted. Intriguingly, however, Blake & Bülthoff [20] noted informally that under monocular viewing, the addition of a specular highlight seemed to bias perception of their stimuli toward convexity, though this effect was not tested empirically. Unlike the binocular effect, such a bias does not have a straightforward geometric explanation. Yet it is important to determine whether this effect is real and quantifiable, since in the real world, disparity signals become very unreliable for distant surfaces, and other visual features (e.g., shading, texture) provide only weak cues to curvature sign. If specular highlights provide a cue to surface convexity, they may prevent observers from making large perceptual errors about distant surfaces in the environment.

Here we ask how specular highlights combine with Lambertian shading cues to determine perceived surface curvature, and to what degree this is based upon a coherent account of the scene geometry. To avoid covariation with other features found in natural scenes (stereoscopic disparity, motion, texture, etc.) we employ relatively simple stimuli (shaded ellipsoidal surfaces with constant albedo), and manipulate the location of highlights relative to the Lambertian shading gradient to vary the consistency of the two cues. In three experiments we ask:

- Does the presence of specular highlights bias the observer toward convex interpretations of monocularly viewed shaded objects?
- How is shape perception affected when highlights are made inconsistent with the Lambertian (smooth) shading of the objects?
- How is the effect of specular highlights on shape perception modulated by surface depth and the slant of the illuminant?

The problem of judging surface shape for these stimuli is ill-posed: there are many possible scene configurations that could give rise to each observed image, and in particular both signs of surface curvature, convex and concave, are possible. Here we hypothesize that the human visual system attempts to determine the most probable curvature sign given the image data. In order to assess whether our psychophysical results are consistent with this principle, we construct a quantitative Bayesian model that attempts to explain the shading and highlights observed in the image in terms of the illumination field, object shape and surface material (glossy or matte). So as not to obscure the empirical results, we defer detailed presentation of the model to the Materials and Methods section, however we will discuss its qualitative properties and show the fit of the model to the psychophysical data alongside our empirical results.

## Results

### Experiment 1

In our first experiment, observers viewed a pair of shaded objects, with or without specular highlights (Figure 1a) and reported perceived sign of surface curvature (convex or concave) of one of the objects. The shading gradients on the two objects were always in opposition and were systematically varied over all angular directions, in 15 deg increments. There were four conditions:

MM: Neither object has a highlight.

SM: The target object has a highlight, the distractor object does not.

MS: The target object does not have a highlight, the distractor object does.

SS: Both objects have highlights.

#### The light from above prior

Figure 1(b–d) show the data and model fit for these four conditions, averaged over observers. The peak of ‘convex’ responses occurred for targets with shading orientations near 0°, i.e. objects that are bright near the top, and darker at the bottom. This peak can be interpreted as the centre of the observer's light prior distribution [2], [25], [26]: observers make an assumption of overhead illumination. This prior over illuminant tilt 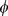 is captured in the model by a von Mises distribution 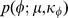 with mean and concentration parameters 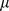 and 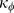 (*Materials and Methods*). The parameters vary across observers, but on average the light prior peaks at almost directly overhead (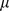 = −3.0±3.8 deg) and is quite broad (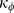 = 7.5±2.3, corresponding to a full width at half-height of 68.9±9.8 deg), all means are reported ±1 standard error of the mean.

#### Highlights increase the proportion of convex responses

Figure 1(b) shows that the appearance of a highlight generally increases the proportion of convex responses. Figure 1(e) summarizes this result by collapsing over all directions of the shading gradient: proportion of ‘convex’ responses varied substantially and significantly across the four highlight conditions (ANOVA with Huynh-Feldt correction: F_(1.15,10.3)_ = 8.39, *p*<0.05). In particular, we found that the presence of a highlight biases observers to report the shape as convex. Observers reported convexity most often when the target object had a highlight, and the other object (the distractor) did not (SM condition; mean proportion ‘convex’ across observers: 64%). In contrast, when the highlight was on the distractor but not the target, the target was more often perceived to be concave (MS condition; mean proportion convex: 46%). In other words, the highlight generates an 18% difference in perceived convexity (*p*<0.05 after corrections for multiple (6) comparisons). The proportion of ‘convex’ responses is also larger in the SS condition than in the MM condition (60% vs. 50%).

We can quantify the effect of a highlight on perceived convexity as a function of the illuminant tilt by calculating the mutual information (MI) of these two variables: how well the presence/absence of the highlight on the target predicts observers' shape responses. Figure 1(f) shows the results, using data from the SM and MM conditions of Experiment 1. The addition of a highlight has little effect when the stimulus is bright at the top (0 deg); stimuli are already perceived as unambiguously convex. However, as the illuminant rotates away from directly overhead, the shape becomes more ambiguous and the effect of the highlight on perceived convexity becomes pronounced, peaking when the direction of illumination is horizontal.

**Figure 2:**
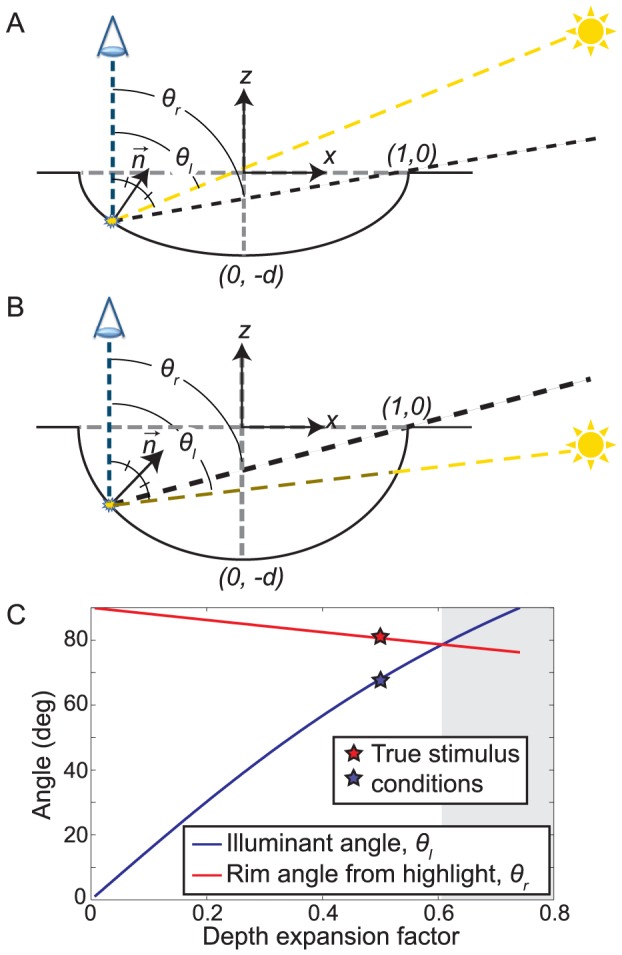
Geometry of specular highlights for concave stimuli. (a) We render our stimuli from a scene geometry in which the angle θ_r_ between the viewing direction and the rim is greater than the angle θ_l_ between the viewing direction and the lighting direction required to produce the visible specular highlight, so that the specular highlight is visible. However, the apparent Lambertian shading pattern is also consistent with the geometry shown in (b), where a deeper surface causes θ_l_ to exceed θ_r_, making the specular highlight infeasible. (c) The blue curve shows the family of (depth, illuminant slant) scene solutions consistent with the rendered image (the bas-relief ambiguity). (The depth expansion factor is the ratio of the depth to the half-width of the hemi-ellipsoidal surface.) The red curve shows how the direction to the rim of the surface changes as the surface depth increases. When the illumination slant exceeds the slant of the rim (shaded region), the light source is occluded, making the appearance of a specular highlight infeasible.

Does this effect have a rational basis? Our hypothesis is that it is rooted in the geometry of self-occlusion. To create our visual stimuli, we set the depth of the object and the slant of the light source (angular deviation from the view vector) so that for a glossy surface a highlight will be visible whether the shape is convex or concave (Figure 2a). However, due to the well-known bas-relief ambiguity [27]–[29], the observer is uncertain not only about the convexity of the surface, but also about the exact surface depth and the light source direction. In the case of a convex surface, this uncertainty does not affect the visibility of the specular highlight: it remains visible regardless of the depth of the surface and the direction of the illuminant. However, the same is not true for a concave surface. In this case, depending upon the depth of the object and the slant of the illuminant, the light ray that would normally generate the specular highlight may be blocked before it can reach the surface (Figure 2b), making the appearance of a highlight impossible.

Mathematical analysis (*Materials and Methods*) reveals a subset of infeasible concave solutions for our stimuli when the depth expansion factor (the ratio of the depth of the object to its half-width) exceeds 0.6, and the slant of the illuminant exceeds 79 deg. These conditions are only modestly beyond the conditions actually rendered (0.5, 68 deg: stars in Figure 2c). In other words, if the observer overestimates the depth magnitude by 20% or more and overestimates the slant of the illuminant direction by 16% or more (i.e. perceives it as closer to the image plane), the image becomes inconsistent with a concave object. Given uncertainty about surface depth and illumination slant, this analysis predicts that the appearance of a highlight should bias observers toward a percept of convexity.

The strength of the bias should depend on the exact family of solutions (surface shape and illumination slant) consistent with the observed stimulus, increasing in strength when the stimulus is altered to be consistent with solutions more likely to cause occlusion of the highlight for a concave surface. In Experiment 3 we explore the effects of varying the stimulus on the convexity bias, however in the current experiment the shading pattern was held constant aside from the angular direction, which does not affect the probability of occlusion. If we knew the internal prior over illuminant slant and surface depth, we could compute the posterior probability that the highlight would be occluded for a concave surface, given the observed shading gradient. Since we do not know these priors, we instead treat the probability of specular highlight occlusion for this stimulus as a free parameter *p_os_*, constrained by the psychophysical data (*Materials and Methods*). The resulting empirical mean of *p_os_*  = 0.38±0.12 constitutes a hard empirical prediction that could in the future be compared to the ecological statistics of illuminant slant and object shape, as well as estimates of these internal priors from psychophysical studies.

**Figure 3:**
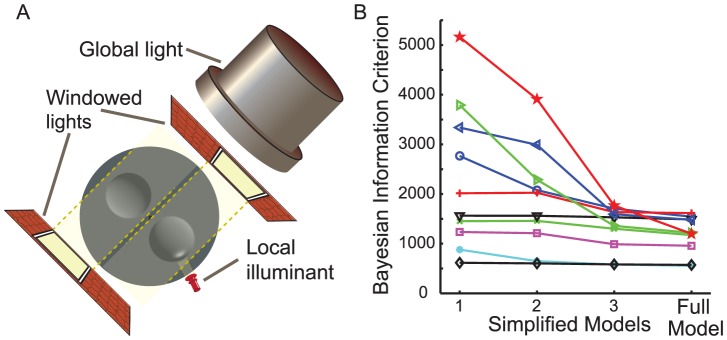
Schematic of illumination configurations and evaluation of simplified models. a) Schematic of the one- and two-light configurations. b) Bayesian Information Criterion for three simplified models and the full Bayesian model (see Table 1 and Figure 7).

In natural viewing, specular highlights and cast shadows are coupled – infeasible specular highlight locations given particular shape and lighting combinations lie inside a cast shadow (although we note that with interreflections it is sometimes possible for a specular highlight to be visible inside a cast shadow). In the absence of uncertainty, therefore, the ‘shiny is convex’ cue would be redundant; if the object's rim casts a shadow, the object is concave. Without a cast shadow, highlights are equally likely to be generated on convex and concave surfaces. However, to be detected, cast shadows must be segregated and distinguished from other luminance modulations such as attached shadows. Given uncertainty about the exact surface shape, this is a challenging task, in part because for non-point light sources, the luminance profile generated by penumbral blur can closely mimic smooth shading due to surface curvature [30], [31]. Indeed, experimental data suggest that shadows cast from local objects do not entirely disambiguate curvature sign [32], [33] and self-shadowing provides only limited improvement in judgements of curvature sign [8]. In the face of these uncertainties, the presence of a specular highlight provides an additional cue to surface curvature that should, based upon our analysis of the geometry, bias observers to perceive the surface as convex. Accounting for the influence of a specular highlight on the perception of surface convexity increases the proportion of variance in the data explained by the model from 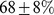 to 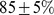 and yields an improved Bayesian Information Criterion (BIC) score (Table 1: model M2 vs. M3, Figure 3b).

**Table 1:** Model parameters.

			Alternative models		
			M1	M2	M3
1	μ	Mean of the prior over the tilt of the main illuminant(s).	✓	✓	✓
2	*κ_φ_*	Concentration of the prior over the tilt of the primary illuminant(s).	✓	✓	✓
3	p(τ_i_ = convex)	Prior for object convexity.		✓	✓
4	p(α = 1)	Probability of a single main illuminant for our stimuli. The probability of two (windowed) illuminants is given by p(α = 2) = 1- p(α = 1).		✓	✓
5	p(χ_i_ = shiny)	Probability that an object is shiny (vs. matte)			✓
6	p_os_	Probability for our stimuli that for a concave object, occlusion by the object's rim makes a specular highlight infeasible.			✓
*7*	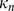	Concentration parameter for von Mises distribution modelling noise in observation of gradient direction and offset of specular highlight from gradient direction.			
8	p(β*_i_* = present)	Probability of a local illuminant. The location is modelled as uniform.			
9	p_om_	Probability that for a concave object, the local illuminant will be occluded.			

#### Relaxing the single light source assumption

The classic shape-from-shading demonstrations of Ramachandran [1], [34] suggest that observers have an internal prior for a single dominant light source, and this assumption is also apparent in our data. On average for the MM condition, observers report 50% of the shapes to be convex and 50% to be concave. Moreover, the probability of convex report for opposite shading directions sums to almost exactly one for each direction, indicating that observers generally see the two oppositely shaded objects as having opposite surface curvature, consistent with a single light source.

However when one or both of the objects has a highlight, the single-light source assumption no longer dominates perception: the probability of convex report for opposite shading directions sums to more than one (1.1 for SM + MS, 1.2 for SS), indicating that on some proportion of trials observers see both shapes as convex.

This interpretation is only possible if the two objects receive different illumination. Such situations occur frequently in natural scenes, due to the complexity of illumination and shadowing, and the causes are often distal, and not necessarily in the field of view. For example, peripheral objects may cast shadows or interreflections on selected parts of the scene.

An ideal Bayesian observer would marginalize over the collection of possible complex scene arrangements that could produce the opposing shading gradients, but implementing this in our model is of course not feasible. Following a minimum description length principal [35], [36] the marginal over all of these more complex scenes can be loosely approximated by the probability of the simplest configuration consistent with the data. In this spirit, our model represents this complex set of illumination fields as two distinct *windowed* illuminants, each acting on only one of the shapes, thus producing illumination fields local to each object (Figure 3a). (In the language of Adelson [37], these might be called local *atmospheres*.) While two independent light fields acting separately on the two objects should be substantially less probable than a single dominant light source, this difference in probability must play off against the observer's prior for convex objects over concave, and any evidence for convexity provided by the highlight.

In summary, our model allows for two different scene configurations: 1) two objects, one convex and the other concave, illuminated by a single global light field, and 2) two objects (which may have the same curvature sign) individually illuminated by two separate, windowed light fields. The prior distribution over the number of illuminants 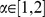 is determined by the single parameter 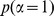 specifying the probability of a single global light field (so that the probability of two windowed illuminants is simply 1 −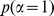, see *Materials and Methods*). The estimated value of this parameter varies over observers, with a mean of 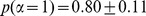. In addition, for the two illuminant solution to have non-vanishing probability, we must assume some uncertainty in the estimation of the gradient direction. This uncertainty is modelled by a von Mises distribution centred on the true gradient direction, with a single concentration parameter 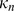. Incorporating these two alternative lighting configurations into the model yields a 17% improvement in the Bayesian Information Criterion score of the model (Table 1: model M1 vs. M2, Figure 3b).

### Experiment 2

**Figure 4:**
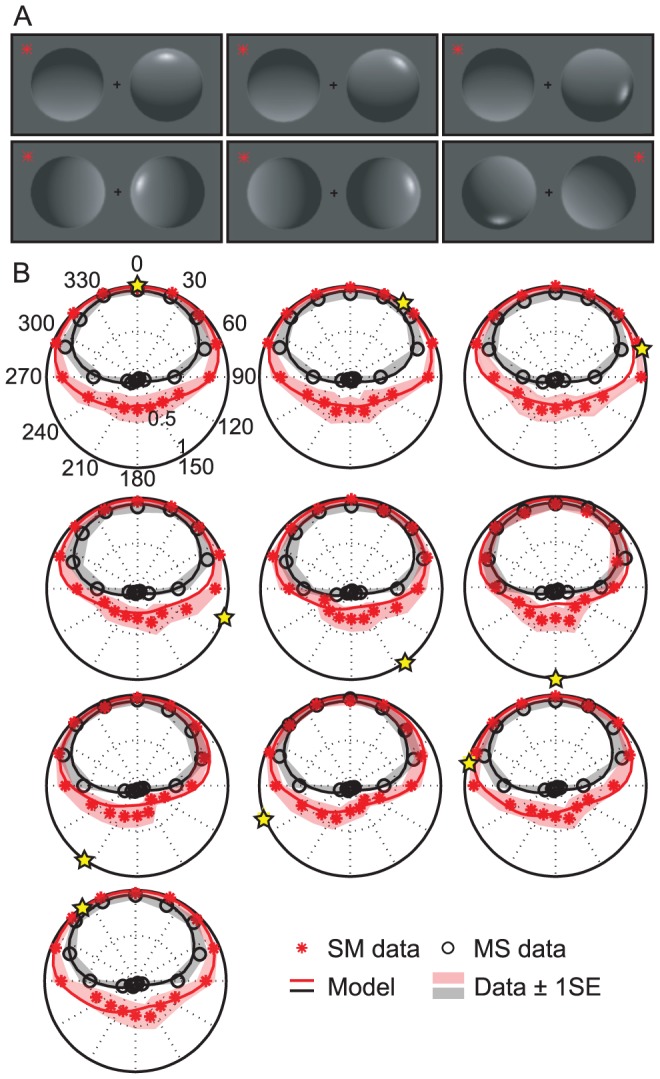
Experiment 2 stimuli and data. (a) 6 examples of the 120 stimulus configurations. For the stimuli in the left and middle columns, most observers will perceive the highlight as a specularity on a shiny object. However, the misaligned highlights in the rightmost column are more often perceived as the result of a local patch of illumination on a matte object. (b) Data averaged across observers. The yellow star indicates the polar angle of the highlight. Black circles and red stars give data for objects with and without a highlight, respectively. Solid lines show the model fit and shaded regions indicate ±1SEM.

Our first experiment shows that the appearance of a specular highlight biases observers toward a convex interpretation of the stimulus. For these stimuli, the geometry of reflection dictates that the highlight appears on the lighter side of the shape, aligned with the shading gradient. In Experiment 2 we ask how the effect on perceived convexity varies as a function of this alignment (Figure 4a).

Figure 4(b) shows data averaged across 10 observers. Each subplot shows perceived convexity as a function of shading orientation for a single specular highlight position (indicated by a yellow star). As in Experiment 1, objects with a highlight were judged to be convex more often than objects without. Furthermore, this effect seems to persist even when the highlight is rotated out of alignment with the shading gradient, although the magnitude of the effect is reduced.

**Figure 5:**
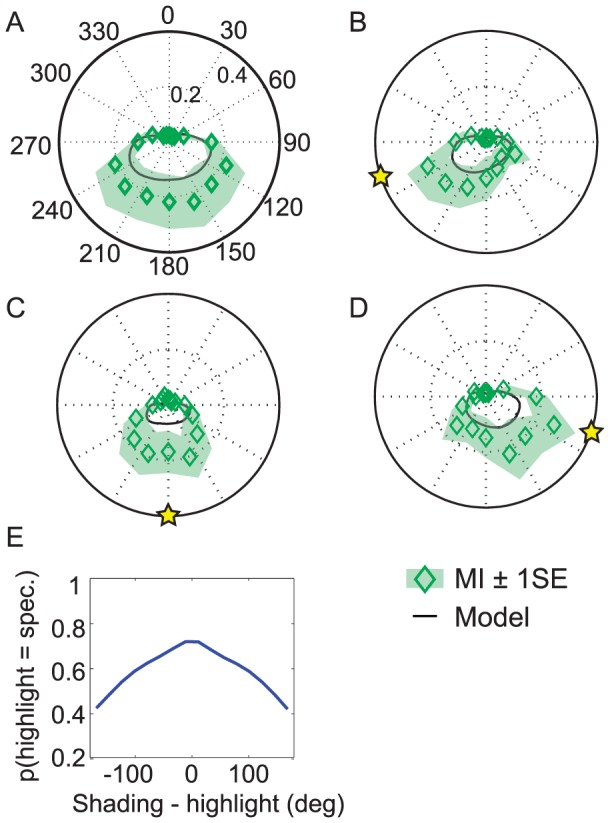
Experiment 2 highlight analyses. Mutual information (MI) between the presence of a highlight and the reported sign of surface curvature, as a function of the shading and highlight orientation. As this analysis is only possible for observers whose perception is modulated by a highlight, we weight each observer's data by his/her MI(highlight, shape) over both experiments. Green diamonds indicate weighted average over observers, and shaded region indicates ±1SEM. The black line indicates the model fit. (a) MI(highlight, shape) as a function of shading orientation, averaged over highlight location. The highlight only has influence when the illumination is not directly overhead. (b–d) MI(highlight, shape) for three example highlight locations. Mutual information is generally highest when the highlight is consistent with the shading. (e) Variation in the probability assigned by the model to the specular interpretation of the highlight (vs. the local illuminant interpretation) as a function of the angular offset between the highlight and shading gradient direction.

To better understand this variation, we calculated the mutual information between the presence of a highlight and perceived shape using data from the SM and MS conditions. Figure 5a shows the results as a function of Lambertian shading orientation, averaged across highlight location. As in Experiment 1, a highlight is ineffectual when objects are bright at the top; these objects are perceived as convex (and lit from above) with or without a highlight. When a highlight appears near the top of the object, therefore, it is not possible to assess whether highlight-shading alignment (and thus highlight interpretation) modulates the effect of highlights on shape perception. However, we can examine the effects of highlight misalignment on shape by considering the mutual information between perceived and convexity and highlights appearing on the lower half of the object (Figure 5(b–d)). We see that the effect on shape is largest when the highlight is aligned, or nearly aligned, with the diffuse shading gradient.

#### Local illuminants

How can these effects be understood in terms of the underlying scene variables? Phenomenologically, as the highlight and shading become misaligned, the surface transitions from glossy to matte in appearance (see Figure 4a and [22], [38]–[42] for related demonstrations), such that the highlight is no longer perceived as a specularity. Previous studies have suggested that in such cases the highlight may be interpreted either as a local change in albedo [38]–[42] or as a region of more intense illumination (e.g. from a spotlight) [38]–[40], although these interpretations have not been tested empirically.

Based on our own impressions and reports from our naïve subjects, for our stimuli the misaligned highlight tends to appear more as a local illumination effect than a variation in albedo. We believe this is largely due to the blurred boundaries of the highlight, more consistent with a change in illumination than in albedo, but it may also be due in part to the relative simplicity of the stimulus. In prior work, Anderson and colleagues [40]–[42] have shown that when the detailed highlight pattern generated from a more complex surface is manipulated to be misaligned with the shading, the incongruence seems to be perceptually explained as a variation in albedo rather than illumination. An example generated by Todd et al [39] (Figure 4) may be considered a middle ground: both the surface and the highlight pattern are of intermediate complexity, and the percept, as the authors report, is ambiguous, “perceptually interpreted as stray beams of light or patches of white paint”.

In our model, a highlight that deviates from the location consistent with the shading gradient can be explained by two alternative scene configurations: (i) A single light source illuminating a shiny surface to produce both the shading gradient and a specularity, displaced due to unmodeled surface variations and/or visual noise, or (ii) A matte surface and a secondary localized illuminant, such as a spotlight, producing the highlight (see Figure 3a). For brevity we will refer to this secondary light source as a *local illuminant*, although in fact the highlight could be produced by other illumination effects, such as dappling from a distant source.

Note that, as for the specular highlight, the local illuminant might also be subject to occlusion for concave objects, although the statistics of this second occlusion effect are likely to be different from those for specular highlights. Thus, even when the highlight is rotated out of alignment with the shading gradient, and is no longer perceived as a specularity, it may still influence the perception of convexity. Our model accounts for this with a second occlusion parameter *p_om_*, representing the probability that the local illuminant will be occluded when the surface is concave. When fit to the data, our model yields an average for this parameter of *p_om_* = 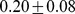 over observers, roughly half the probability of specular occlusion (*p_os_* = 0.38±0.12). We note that as either the specular highlight or the local illuminant might be occluded, this leads to additional possible scene configurations under the concave shape interpretation, namely that the surface is glossy *and* there is a local illuminant *and* either the specular highlight or the local illuminant (but not both) are occluded. These possibilities are fully accommodated by our Bayesian model.

The balance between these alternative scene explanations is governed by the prior probability 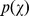 over the specular index 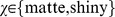 as well as the prior probability 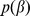 over the appearance of a local illuminant 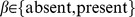 and the angular misalignment 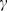 of the highlight with the shading gradient. The model fit to the psychophysical data yields estimates of 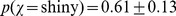 and 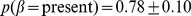 over observers. The highlight misalignment, 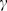, modelled as a 0-mean von Mises distribution, using the same concentration parameter 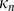 used to model uncertainty in the direction of the shading gradient has an average concentration over all 10 observers of 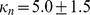, corresponding to a full width at half-height of 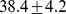 deg.

We assume that a highlight generated by a local illuminant may occur anywhere on the shape with uniform probability. However, a highlight generated by specular reflection is more likely for smaller misalignments between highlight and shading gradient. The effect of this von Mises prior for the specular highlight is a modulation of the posterior probability that the surface is specular as a function of the angular displacement of the highlight from the shading gradient (Figure 5e). The probability of a specular interpretation peaks at 0.72 (averaged across observers, weighted by MI(shape, highlight)) when the highlight is in perfect alignment with the shading gradient, and descends to 0.42 when the highlight is maximally unaligned, i.e., when it appears in the dark area of the shaded ellipsoid.

Allowing for the alternative local illuminant account of the highlight significantly increases the proportion of variance in the data explained by the model, from 85±5% to 90±2% and improves the Bayesian Information Criterion score by 15% (Table 1: model M3 vs. full model, Figure 3b).

### Experiment 3

The specular occlusion account is qualitatively consistent with the observed bias to convex surfaces induced by the appearance of a highlight, but without quantitative measurement of the prior over object shape and illuminant slant it cannot be verified quantitatively. Here we present an additional psychophysical experiment that provides an additional test of the model.

The specular occlusion hypothesis is rooted in uncertainty over the exact shape of the surface and the location of the illuminant. As a result, visual cues that shift the posterior distribution over these scene variables should alter the probability of highlight occlusion and therefore the induced convexity bias. In particular, the bias should get stronger when these cues suggest either (i) an increase in surface depth or (ii) an increase in illuminant slant (deviation from the view vector), since both variations increase the probability of specular occlusion for a concave surface.

**Figure 6:**
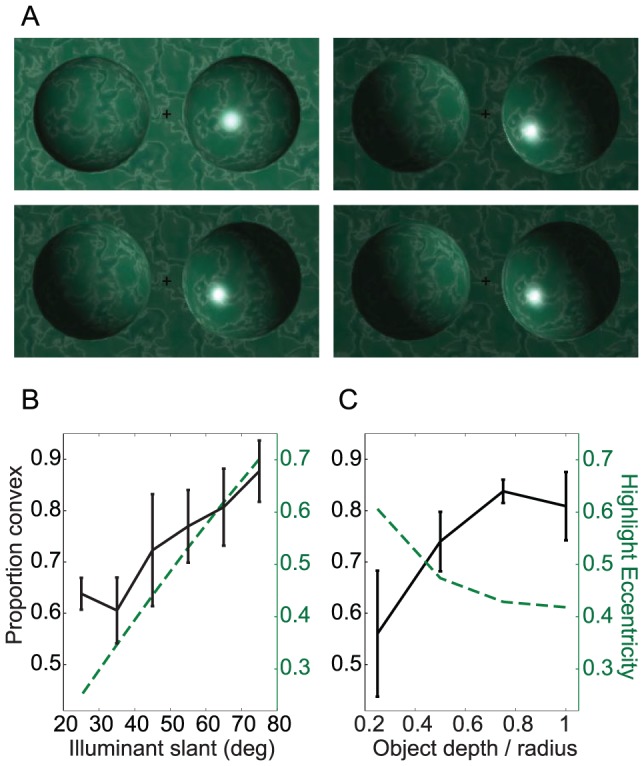
Experiment 3. (a) 4 examples of the 336 stimulus configurations. In the left column, object depth is fixed (depth  =  ±0.75× half-width) but illuminant slant varies between the top (25°) and bottom (55°) images. In the right column, illuminant slant is fixed (65°) but object depth varies between the top (0.5) and bottom (1) images. Further stimulus examples can be found in Figure S2. (b) The effect of adding a highlight on the perception of surface curvature sign, as a function of illuminant slant, averaged across object depth. The dashed green lines in (b) and (c) give the highlight eccentricity, i.e. distance from the object's centre/object radius. (c) The highlight effect as a function of object depth, averaged across illuminant slant. The data are averaged across the four observers. Error bars indicate ±1SEM.

Our third experiment directly tests this prediction of the model (we thank one of the anonymous reviewers for suggesting such an experiment). As in Experiments 1 and 2, observers viewed pairs of shaded stimuli, and reported the perceived shape (convex or concave) of one object. The shading and highlight cues to absolute depth are subtle and confounded with illuminant slant; by adding texture to the objects we provided an independent cue to depth that should allow observers to better dissociate these two scene variables (Figure 6a). The shading gradients of the two objects were always in opposition and either one or neither of the objects had a specular highlight. The two objects always had the same depth magnitude, however, this depth and the slant of the illuminant varied across trials.

To focus the experiment, we determined the shading gradient direction for each observer that produced balanced (50%) reports of ‘convex’ and ‘concave’ for the two oppositely shaded matte objects, and then examined the effect of the highlight on perceived convexity while varying object depth and illuminant slant.

Figure 6 shows example stimuli and the data from this experiment. The highlight effect is quantified by the proportion of ‘convex’ responses in the presence of a highlight (in contrast to 50% when absent). Figure 6b shows the effect as a function of illuminant slant, collapsed across stimulus depth. As the direction of illumination approaches the image plane (increasing slant), the effect of the highlight on perceived shape increases (F_5_ = 7.3; p<0.01). Figure 6c shows the effect as a function of stimulus depth, collapsed across illuminant slant. As object depth increases, the effect of the highlight on perceived shape again increases (F_3_ = 6.2; p<0.05). In summary, as predicted by the geometry of specular occlusion, increases in illuminant slant or object depth both *increase* the probability of convex report.

Interestingly, while increasing illuminant slant or object depth both increase the convexity bias, they have opposite effects on the position of the highlight (dashed lines in Figures 6b and c). In particular, while increasing the slant of the illuminant shifts the highlight toward the rim of the object, increasing the depth of the object shifts the highlight in the opposite direction, toward the centre of the object. Our results therefore indicate that the observer is not simply relying on the position of the highlight when judging curvature sign. Instead, our data suggest that the observer's perception is modulated by estimates of quantitative depth and illumination direction, becoming increasingly biased toward a convex interpretation as the probability of highlight occlusion increases. These results are thus a strong confirmation of the specular occlusion account of the convexity bias induced by the appearance of a highlight.

## Discussion

We have conducted three experiments to explore the effects of highlights on perceived convexity:

- Experiment 1 demonstrated a clear effect of the presence of a highlight on shape perception – objects with a highlight are more likely to be perceived as convex. The effect is greatest when the shading gradient is horizontal and the sign of surface curvature is most uncertain.
- Experiment 2 explored the role of the alignment between the highlight and the Lambertian shading. Prior work suggests that as the two become misaligned, the highlight is no longer perceived as a specularity [22], [40]–[42]. Our results show that misalignment also reduces (but does not eliminate) the convexity bias, consistent with the interpretation that specular highlights are less likely to be visible on concave surfaces.
- Experiment 3 provided an independent test of the specular occlusion hypothesis. In particular, the results show that increasing the probability of specular occlusion by either a) increasing the depth of the object or by b) increasing the slant of the illuminant increases the convexity bias. Importantly, these results cannot be explained simply by the position of the highlight on the object, strengthening support for an account based on the probability of specular occlusion and rooted in the 3D geometry of the scene.

The results from all three experiments are consistent with a Bayesian model that takes into account potential light source occlusion. Does this mean that observers are constructing a complete and detailed 3D solution for the entire scene? Some have argued against this kind of ‘inverse optics’ model [14], suggesting that the underlying variables of shape, reflectance and illumination may not be estimated concurrently, so that probing the percept of each will not necessarily yield consistent results. Furthermore, while shape and material may be important for manipulating and recognizing objects, we might question whether observers require an explicit estimate of the illumination field.

On the other hand, there is evidence that observers make judgments of shape and/or reflectance consistent with a particular estimate of the illumination field without necessarily making this estimate explicit. Observers can manipulate the shading pattern of one object to appear consistent with a second object, such that the implicit illumination environments match [43], although like our observers, they relied on priors for overhead illumination and object convexity when image cues were ambiguous. Similarly, reflectance judgements for ambiguous images are consistent with a single overhead illuminant [25]. In contrast, observers are poor at making explicit judgements of illumination consistency across multiple objects [44].

In our experiments, observers are asked only to judge the convexity of objects, and not the glossiness of the surfaces or the number or direction of light sources. As a consequence, the predictions of the Bayesian model (*Materials and Methods*) are not based upon explicit joint estimation of these scene variables, but do depend critically on at least approximate marginalization over the unknown ‘nuisance’ variables (object depth, illumination) when judging convexity. This process of marginalizing over or ‘integrating out’ nuisance variables when judging other scene variables of interest is widely believed to explain a number of visual phenomena (e.g., [27], [45]), and the consistency of our Bayesian model with the psychophysical data suggests that it may also explain the effect of highlights on the perception of surface convexity.

The interplay between the light field, surface reflectance and surface shape is complex and many issues remain to be resolved. Our experiments reveal the effect of specular highlights on perceived convexity for ellipsoidal surfaces and point light sources. It remains to be seen whether this effect generalises to more complex surfaces and light fields (see Figure S1 for examples of ellipsoidal stimuli rendered with ray-tracing under a complex illumination field). In addition, further studies may resolve the existing inconsistencies in the literature regarding the effect of highlights on perceived curvature magnitude [8]–[14].

Overall, our results shed new light on how the human brain uses highlights to disambiguate 3D surface shape. Our Bayesian model suggests that this is more than a ‘bag of tricks’[46]. Rather, inference can be accounted for as a rational computation that selects the most probable shape interpretation, given the observed data and prior information about the relative probability of alternative scene configurations.

## Methods and Model

### Ethics

For all experiments, participants gave informed consent and the local ethics committee approved the study.

### Methods experiment 1

Stimuli consisted of two axis-aligned half-ellipsoids, compressed in depth by a factor of two relative to a hemisphere, illuminated by a single, distant light-source. The orientations of the smooth (Lambertian) shading gradients on the two objects were always in opposite directions.

When a single object (either with or without a highlight) is presented in isolation it is perceived as convex for all illumination tilts due to the widely documented prior for object convexity [47]–[50]. This convex bias is represented in our model by the prior over curvature sign 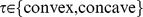: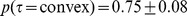 over observers. When two objects are presented with opposing shading gradients, the prior for a single illuminant counteracts the convexity prior, causing the observer to perceive the objects as having opposing curvature sign, on most trials. The two-object scene thus allows us to explore the effects of specular highlights on shape perception.

There were four stimulus configurations: (1) Highlight on neither object, (2) Highlight on the left object, (3) Highlight on the right object, (4) Highlight on both objects (Figure 1a). Stimuli were generated as grey objects under white light using the Phong lighting model implemented in OpenGL, without inter-reflections or cast shadows, under orthographic projection. Shiny objects were rendered with ambient (7% of maximum), diffuse (36% of maximum) and specular components (48% of maximum, with Phong exponent of 80). Matte objects had only diffuse and ambient components.

Under this Phong lighting model and orthographic projection, convex and concave objects generate identical images, thus rendering the estimation of the sign of surface curvature completely ill-posed, allowing us to isolate the role of highlights in the perception of surface convexity. In a real scene, however, subtly different patterns of interreflection could in theory serve to discriminate convex from concave surfaces. In practice however, these differences are relatively minor for our scenes, as confirmed by comparing ray-traced renderings, under a complex light field, with and without inter-reflections (compare Figures S1a and b).

We define a coordinate frame with origin at the centre of the display, X- and Y-axes in the horizontal and vertical directions in the plane of the screen, respectively, and Z-axis positive toward the observer. The slant of the single directional light (the angle between the lighting vector and the Z-axis) was held constant at 68°. The tilt of the lighting direction (the angle between the projection of the lighting vector and the Y-axis) varied across trials. The orientation of the shading gradient for each object was thus a function of its curvature sign and light source tilt. The room was unlit aside from the light emitted by the monitor. To eliminate binocular and motion-based depth cues, stimuli were viewed monocularly, with the observer's head fixed by a chin rest and forehead bar. At the viewing distance of 57 cm, each object subtended 5° with their centres displaced horizontally ±3.4° from the display centre. Scenes were rendered with orthographic projection, simulating an infinite viewing distance. Given the small angular subtense of our stimuli, switching to perspective projection has only a small effect on the shading gradient and position of the highlight in our images (see Figure S1c).

On each trial, the two shaded objects appeared for 1 second. Halfway through the presentation, a star appeared next to one of the objects, indicating that this ‘target’ should be judged. By a key-press, the subject reported the target curvature as either ‘convex’ or ‘concave’. The four conditions (Target Matte, Distractor Matte (MM); Target Matte, Distractor Shiny (MS); Target Shiny, Distractor Matte (SM); Target Shiny, Distractor Shiny (SS)) and the target's shading orientation were randomly interleaved. Ten observers (9 naïve and 1 author) each completed 1536 trials (24 target orientations x 4 conditions x 16 repetitions) in a single session lasting approximately 1 hour. One additional naïve observer was excluded from the analyses as the direction of the shading gradient had little effect on his/her shape judgements.

### Methods experiment 2

Only one of the two objects was rendered with a highlight, and the orientation of the diffuse shading component (16 equally spaced values) and the angular position of the highlight (10 equally spaced values) were varied independently, by rendering the diffuse and specular components of the image with independently positioned illuminants (Figure 4). As in Experiment 1, the two objects had opposite gradient directions and a star indicated which of the two objects should be judged (convex vs. concave). The 3840 trials (10 highlight positions x 16 shading orientations x 2 conditions (SM: only the target has a highlight, MS: only the distractor has a highlight) x 12 repetitions) were completed in 3 sessions of approximately 45 minutes. All other details were identical to Experiment 1. The 10 observers who completed Experiment 1 also participated in Experiment 2.

### Methods experiment 3

In our third experiment we studied the effect of object depth and illuminant slant on the convexity bias caused by a highlight. This is tricky to do in a controlled fashion using the ellipsoid objects of Experiments 1 and 2, as the variation in curvature across the shape induces changes to both the shape and size of the highlight as the slant of the illuminant is varied. To stabilize the appearance of the highlight, we replaced the ellipsoidal surfaces with sections of hemispheres that protruded from or recessed into the planar background surface. Since surface curvature is constant over the hemisphere, variations in illuminant slant induce much smaller variations in the shape and size of the highlight.

As in Experiments 1 and 2, the direction of the shading gradient on the two objects was always in opposition, such that the stimulus was consistent with one convex and one concave object, both illuminated by a single light source. The simulated depth of both objects always matched, but varied across trials (depth:radius ratio was 0.25, 0.5, 0.75 or 1), by changing the radius of the sphere from which the domes were constructed. Highlight position was yoked to the shading gradient: i.e. both were rendered with the same illuminant.

In order to determine the shading gradient that produces a roughly balanced perception of convex and concave shape for each object depth and illuminant slant, for each observer, we sampled a range of illumination tilts between 90° to 270° (7 equally spaced values). Illuminant slant varied across trials from 25° to 75° (6 equally spaced values). A green texture (see Figure 6a) was wrapped around both objects and the planar background to facilitate depth perception. We found that the sharp join between the hemisphere sections and the planar background caused the objects to appear detached from the background; to avoid this, we introduced a thin curved section to smooth this join. For specular objects, this generated an additional very thin specularity at the join (Figure 6a); this additional feature does not appear to be correlated with variations in observer reports of perceived convexity. Four observers completed 2016 trials (4 depths x 6 illuminant slants x 7 illuminant tilts x 2 specularity conditions (no highlights or highlight on the target only) x 6 repetitions in two sessions of approximately 30 minutes.

Both object depth and illuminant slant have a systematic effect on the perceived curvature sign of matte objects; shallow objects and small illuminant slants produce shading patterns that are more similar for the two objects, and perhaps for this reason the overall proportion of convex responses increases under these conditions (although this did not reach significance). To compare the effect of the highlight across these conditions without the confound of varying baseline convexity, for each condition and each subject we found the shading orientation at which the matte stimulus was perceived as convex on 50% of trials. This was found by fitting a psychometric function to the proportion of convex responses as a function of shading orientation, and obtaining the 50% threshold. We then measured the effect of the highlight by the proportion of convexity judgments relative to this consistent 50% baseline (Figures 6b and c).

### Model

Our psychophysical experiments have shown that the judgement of surface convexity is dependent upon the appearance of surface highlights and their locations relative to the shading gradient induced by surface curvature. In our view, the most important question is *why* a highlight has this effect. Here we put forward a specific theory: due to potential occlusion of the light source for a concave surface, highlights occur more frequently on convex surfaces in natural scenes. As a consequence, the convexity bias induced by highlights will increase the ability of the observer to correctly judge the sign of surface curvature.

**Figure 7:**
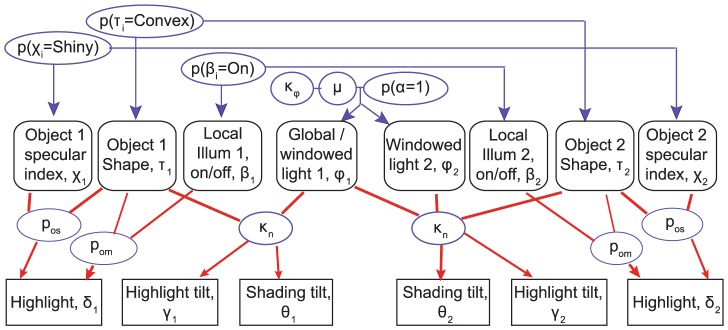
Graphical representation of the model. Shown are the observable variables (rectangles), generative object and illumination components (rounded rectangles) and the model's 9 free parameters (ellipses).

While this theory is qualitatively consistent with the psychophysical data, it remains to be seen whether it is quantitatively consistent with the data. To assess this, we have constructed a Bayesian model for the discrimination of convex vs concave surface curvature given the shading gradients and highlights appearing on the two objects comprising our stimuli. Specifically, the observable variables are (Figure 7):

- the tilt of the smooth shading on each object (θ_1_ and θ_2_)
- the absence or presence of a highlight on each object (δ_1_ and δ_2_)
- the tilt of the highlight on each object (γ_1_ and γ_2_)

The model incorporates the minimal set of hidden scene variables sufficient to explain the observed shading and highlight cues. These include:

1. **The number of light sources generating the shading gradients.** Our observers generally perceive objects with oppositely oriented shading gradients as illuminated from a single direction but having opposite curvature. However, for roughly horizontal directions, and particularly in the presence of a highlight, observers often perceive both objects as convex. The only way to account for this is to allow for a more complex lighting model that illuminates the two different objects from opposite directions. This is captured in the model by the variable 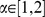 specifying the number of illuminants, and the prior 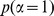 specifying the probability of a single global light field.
2. **The tilt** 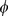 **of each of the light sources.** These are necessary to explain the directions of the shading gradients. The light from above prior is modelled as a von Mises distribution 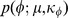.
3. **The specular index of each shape.** In agreement with previous reports [22], [38]–[42], under some conditions the presence of the highlight makes the shape look shiny and under others it does not. This necessitates a variable 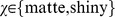 that codes the specular index of the shapes and a prior 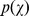 over this variable.
4. **The number of local illuminants.** When the highlight location is inconsistent with the gradient direction, the shape generally looks less shiny, in which case the highlight must have an alternate explanation. Possibilities include a local increase in albedo (‘paint’) and a local increase in illumination (see Figure 3a). From our own observations and informal reports from naïve subjects, the misaligned highlight in our stimulus generally appears as the latter. This necessitates a variable 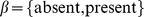 that codes the presence of a local illuminant that can account for the highlight, and a prior 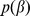 over this variable.
5. **Object shape (convex or concave).** This is what the observer reports. We represent this with the variable 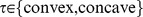 and a prior 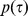 over this variable captures the general bias to see shape as convex.

We believe this to be the minimal set of hidden variables that makes sense: removal of any one of these variables would mean that the model would not capture a basic feature of the phenomenology or relationship between observable features and observer reports (see *Model complexity*).

Capturing the relationship between perceived surface curvature sign and illumination requires modelling probability distributions over the angular direction (tilt) of the illuminant and corresponding observable variables. Observers have a well-documented prior for overhead illumination [1], [2], [25], [26], [34], [48], [51]–[53] that has previously been successfully modelled by a von Mises distribution [51] although the mean of this distribution varies considerably across observers [25]. We employ the von Mises distribution to model observers' prior distribution over illuminant tilt, with the general form
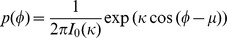
where 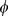 is the tilt angle, 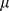 and 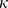 are the mean and concentration (inverse variance), and 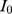 is the modified Bessel function of order 0, required for normalization. This distribution is used to model:

1. The prior distribution 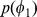 and 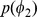 over tilt of the primary illuminants.
2. The likelihood distributions 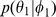 and 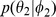 for the observed tilt of the shading gradient, given the tilt of the light source. The mean of the distribution over 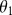 and 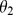 are 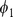 and 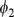, respectively, for convex objects and 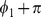 and 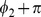, respectively, for concave objects. However, due to noise in the visual estimation of the gradient direction, as well as uncertainties in the exact surface shape, the estimated values 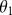 and 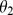 will deviate randomly from these expected values.
3. The likelihood distributions 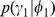 and 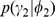 for the observed tilt of the specular highlight, given the tilt of the light source. As for the shading gradients, the mean of the distribution over 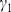 and 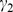 are 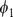 and 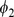, respectively, for convex objects and 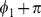 and 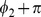, respectively, for concave objects.

For each observer, the values of the 9 model parameters (summarized in Table 2) were found (MATLAB fminsearch) that maximize the joint likelihood of the observed data for both Experiments 1 and 2. Multiple iterations of the parameter search were performed, with the initial values on each iteration determined by uniform sampling within a plausible parameter range. All equations for the model can be found in Text S1.

**Table 2:** Maximum likelihood parameter estimates for the full model for individual observers.

Obs	μ	*κ_φ_*	p(τ_i_ = convex)	p(α = 1)	p(χ_i_ = shiny)	p_os_	*κ_n_*	p(β*_i_* = present)	p_om_
**1**	−0.35	2.5	1.00	1.00	0.41	0.06	14.8	0.88	0.39
**2**	−0.29	3.5	0.82	1.00	0.95	0.40	2.6	0.88	0.00
**4**	−0.36	1.6	0.78	0.94	1.00	0.00	2.0	1.00	0.00
**5**	0.02	20.0	0.98	0.70	0.89	0.51	4.6	0.95	0.09
**6**	0.16	9.7	0.50	1.00	0.00	0.26	2.6	0.90	0.24
**7**	0.02	9.6	0.46	1.00	0.52	1.00	3.5	0.75	0.07
**8**	0.14	4.2	0.56	1.00	0.29	0.53	1.4	0.44	0.13
**9**	0.20	1.6	0.98	0.39	1.00	0.01	3.9	0.95	0.10
**10**	−0.07	20.0	1.00	0.96	0.99	0.06	2.0	1.00	0.17
**11**	−0.01	2.3	0.44	0.00	0.00	1.00	12.3	0.00	0.80
**Mean**	−0.05	7.5	0.75	0.80	0.61	0.38	5.0	0.78	0.20
**SD**	0.21	7.2	0.24	0.34	0.41	0.38	4.7	0.32	0.24

### Model complexity

Our model was constructed to include only scene variables relevant to the observers' judgement of convexity for the two-object stimuli used in our experiments. Nevertheless, the model does have nine free parameters, raising the question of whether we are overfitting the data.

To address this question, we considered three models of reduced complexity and compared their ability to account for the psychophysical data (Table 1).

1. *Model 1*: This model ignores specular highlights and allows for only one, global illuminant, forcing the two objects to be assigned opposite curvature sign. The prior over curvature sign is uniform, i.e. 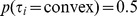.
2. *Model 2*: Here we allow the possibility that both objects in a scene have the same curvature sign, despite opposing shading gradients, by adding the possibility of separate illumination for the two objects, and a non-uniform prior over curvature sign.
3. *Model 3*: This model also allows the appearance of a highlight to influence the perception of surface convexity, through the possibility that highlights may be occluded for concave surfaces. However, any offset of the highlight position, relative to the shading orientation, is ignored; all highlights are attributed to specular reflections rather than variations in local illumination.

We find that the full model provides the best account of the data, for every observer, as indexed by the Bayesian Information Criterion (see Figure 3b). This result suggests that to account for the perception of surface convexity one must allow for a) a prior bias for convexity, b) the possibility of complex illumination fields, c) the biasing effects of highlights and d) the possibility of attributing these highlights either to specular reflection or to a local illumination effect, depending upon the consistency of the highlight with the shading gradient.

### Occlusion geometry

To understand the scene parameters leading to specular highlight occlusion, we can, without loss of generality, consider the viewing geometry of our scene in cross-section, in the plane defined by the viewing and illuminant vectors, with the illuminant on the right (see Figure 2a). The resulting cross-section of the surface describes a semi-ellipse. We define the depth expansion factor *d* to be the ratio of the length of the semi-axis in the viewing direction *z* to the length of the semi-axis in the horizontal direction *x*. Without loss of generality, we assume that the length of the semi-axis of the ellipse in the horizontal direction is 1, so that the length of the other semi-axis (in the viewing direction *z*) is equal to the depth expansion factor *d*. Centering a 2D coordinate system directly above the concave surface, at the level of the rim, the surface cross-section can be described by the equation
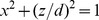
(0.1)


Taking a first derivative yields 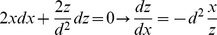, so that the tangent vector 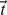 must be in the direction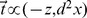 and the normal vector 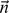 must be in the direction 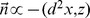.

The specular highlight will be located at the point 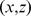 on the semi-ellipse where the normal bisects the angle 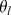 formed by the view vector and the illuminant vector. Thus we have
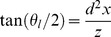
(0.2)


Together, Equations (0.1) and (0.2) determine the location of the highlight: solving (0.2) for 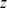 and substituting in (0.1) yields
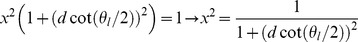
(0.3)


For our stimuli, the depth expansion factor and illuminant direction were fixed at 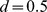 and 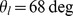, yielding a highlight location of 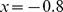.

Of course the observer does not know the exact surface depth or illuminant direction, and for a highlight appearing at this particular location 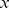 there is in fact a one-dimensional family of solutions 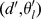 to Equation (0.3) given by
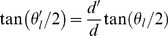
(0.4)and described by the blue curve in Figure 2c.

However, not all of these solutions are physically possible: for larger illumination angles (and larger surface depths), the view of the illuminant from the required highlight location will be occluded by the rim of the surface. To quantify this constraint, we note that the angle 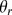 of the vector pointing to the rim from the highlight location, relative to the view vector (Figure 2a), can be written as
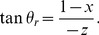
(0.5)


Substituting for 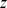 from (0.2) yields



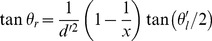
(0.6)and substituting for 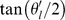 from Equation (0.4) yields
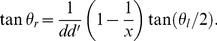
(0.7)


Equation (0.7) describes the angle of the rim of the surface as seen from the potential highlight location, as a function of the *estimated* depth expansion factor 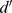. This function is shown by the red curve in Figure 2c. Note that for a subset of solutions with highly oblique illumination and large surface depth, the red curve lies below the blue curve. These solutions are physically infeasible because the illuminant is occluded by the rim of the surface.

For a Bayesian observer who is uncertain about the surface depth and elevation of the illuminant, a consequence is that observation of a highlight will decrease the probability of concave surface curvature relative to the probability of convex surface curvature, for which all solutions are feasible.

## Supporting Information

Figure S1 **Stimuli rendered under complex light field.** a) The stimuli used in Experiments 1 and 2, but rendered via ray-tracing (with multiple bounces) as glossy surfaces under a complex illumination field (an HDR light field captured in a forest environment). The object on the left is convex and the object on the right is concave. Note that under these more realistic rendering conditions (that include faint cast shadows) some ambiguity in curvature sign remains. Readers who correctly perceive the curvature sign of the two objects should rotate the image (or themselves) by 180°; the concave object is now likely to appear convex. b) The same as (a), with inter-reflections removed. The difference is subtle. c) The same as (a), but under perspective, rather than orthographic projection. Again, the difference is subtle. Readers should note that many highlights appear on both left and right objects in (a–c). Some of these highlights are equally consistent with convex and concave shape interpretations: they are consistent with object shapes and illuminant directions that do not approach the conditions for highlight occlusion, and thus should not bias perceived convexity. However, other highlights will be consistent with scene values (illuminant direction and quantitative shape) that produce occlusion for concave objects, and should thus bias perception toward convexity. This predicts that the effect of a highlight on perceived curvature will be jointly determined by the location of the highlight and the estimated object shape, and this prediction is borne out in the results of Experiment 3. d) The same as (a) but with matte surfaces. Anecdotally, we find that the matte concave object looks more reliably concave than do the glossy concave objects.
(EPS)

Figure S2 **Examples of stimuli used in Experiment 3.** In these examples illuminant tilt is fixed at 210°. Within each column, illuminant slant increases from 25° to 75° (6 equally spaced values). (a) The objects have a depth:half-width ratio of 0.5, and the target object has a highlight. (b) As in (a), but both objects are matte. (c) As in (a) but objects have a depth:half-width ratio of 1.
(EPS)

Text S1 **Model equations: The equations for the full model.**
(PDF)

## References

[1] Kleffner D, Ramachandran V (1992) On the perception of shape from shading. Perception & Psychophysics 52: 18–36. 1635855 10.3758/bf03206757

[2] Adams W, Graf E, Ernst M (2004) Experience can change the ‘light-from-above’ prior. Nature Neuroscience 7: 1057–1058. 15361877 10.1038/nn1312

[3] Kingdom F (2003) Color brings relief to human vision. Nature Neuroscience 6: 641–644. 12740582 10.1038/nn1060

[4] Longuet-Higgins MS (1960) Reflection and refraction at a random moving surface (I) Patterns and paths of specular points. Journal of the Optical Society of America 50: 838–844.

[5] Weidenbacher U, Bayerl P, Fleming R, Neumann H (2005) Perception of mirrored objects: A modeling approach. Perception 34: 177–177.

[6] Koenderink JJ, Vandoorn AJ (1980) Photometric invariants related to solid shape. Optica Acta 27: 981–996.

[7] Fleming R, Torralba A, Adelson E (2004) Specular reflections and the perception of shape. Journal of Vision 4: 798–820. 15493971 10.1167/4.9.10

[8] Liu B, Todd J (2004) Perceptual biases in the interpretation of 3D shape from shading. Vision Research 44: 2135–2145. 15183680 10.1016/j.visres.2004.03.024

[9] Todd J, Mingolla E (1983) Perception of surface curvature and direction of illumination from patterns of shading. Journal of Experimental Psychology-Human Perception and Performance 9: 583–595. 6224894 10.1037//0096-1523.9.4.583

[10] Curran W, Johnston A (1996) The effect of illuminant position on perceived curvature. Vision Research 36: 1399–1410. 8762759 10.1016/0042-6989(95)00213-8

[11] Norman JF, Todd JT, Orban GA (2004) Perception of three-dimensional shape from specular highlights, deformations of shading, and other types of visual information. Psychological Science 15: 565–570. 15271003 10.1111/j.0956-7976.2004.00720.x

[12] Mingolla E, Todd J (1986) Perception of solid shape from shading. Biological Cybernetics 53: 137–151. 3947683 10.1007/BF00342882

[13] Nefs H, Koenderink J, Kappers A (2006) Shape-from-shading for matte and glossy objects. Acta Psychologica 121: 297–316. 16181604 10.1016/j.actpsy.2005.08.001

[14] Khang B, Koenderink J, Kappers A (2007) Shape from shading from images rendered with various surface types and light fields. Perception 36: 1191–1213. 17972483 10.1068/p5807

[15] Ho Y-X, Landy MS, Maloney LT (2008) Conjoint measurement of gloss and surface texture. Psychological Science 19: 196–204. 18271869 10.1111/j.1467-9280.2008.02067.x PMC2679902

[16] Hartung B, Kersten D (2002) Distinguishing shiny from matte. Journal of Vision 2: 551.

[17] Doerschner K, Fleming RW, Yilmaz O, Schrater PR, Hartung B, et al (2011) Visual Motion and the Perception of Surface Material. Current Biology 21: 2010–2016. 22119529 10.1016/j.cub.2011.10.036 PMC3246380

[18] Hurlbert AC, Cumming BG, Parker AJ (1991) Recognition and perceptual use of specular reflections. Investigative Ophthalmology & Visual Science 32: 1278–1278.

[19] Hurlbert A, Cumming B, Parker A (1992) Local cues from specular highlight motion influence global shape perception. Perception 21: 1–114.

[20] Blake A, Bülthoff H (1990) Does the brain know the physics of specular reflection? Nature 343: 165–168. 2296307 10.1038/343165a0

[21] Wendt G, Faul F, Mausfeld R (2008) Highlight disparity contributes to the authenticity and strength of perceived glossiness. Journal of Vision 8: 14.1–10 10.1167/8.1.14 18318617

[22] Kerrigan IS, Adams WJ, Graf EW (2010) Does it feel shiny? Haptic cues affect perceived gloss. Journal of Vision 10: 868.

[23] Nefs HT (2008) Three-dimensional object shape from shading and contour disparities. Journal of Vision 8: 11.1–16 10.1167/8.11.11 18831605

[24] Kerrigan IS, Adams WJ (2013) Highlights, disparity, and perceived gloss with convex and concave surfaces. Journal of Vision 13: 9.1–10 10.1167/13.1.9 23291649

[25] Adams W (2007) A common light-prior for visual search, shape, and reflectance judgments. Journal of Vision 7: 11: . 1-7.doi 10.1167/7.11.11. 10.1167/7.11.11 17997666

[26] Adams W (2008) Frames of reference for the light-from-above prior in visual search and shape judgements. Cognition 107: 137–150. 17950264 10.1016/j.cognition.2007.08.006

[27] Freeman W (1994) The generic viewpoint assumption in a framework for visual perception. Nature 368: 542–545. 8139687 10.1038/368542a0

[28] Belhumeur P, Kriegman D, Yuille A (1999) The bas-relief ambiguity. International Journal of Computer Vision 35: 33–44.

[29] Koenderink J, van Doorn A, Kappers A, Todd J (2001) Ambiguity and the ‘mental eye’ in pictorial relief. Perception 30: 431–448. 11383191 10.1068/p3030

[30] Elder J, Zucker S (1998) Local scale control for edge detection and blur estimation. Ieee Transactions on Pattern Analysis and Machine Intelligence 20: 699–716.

[31] Elder J, Trithart S, Pintilie G, MacLean D (2004) Rapid processing of cast and attached shadows. Perception 33: 1319–1338. 15693674 10.1068/p5323

[32] Erens R, Kappers A, Koenderink J (1993) Estimating local shape from shading in the presence of global shading. Perception & Psychophysics 54: 334–342. 8414892 10.3758/bf03205268

[33] Berbaum K, Bever T, Chung C (1984) Extending the perception of shape from known to unknown shading. Perception 13: 479–488. 6527935 10.1068/p130479

[34] Ramachandran VS (1988) Perceiving shape from shading. Scientific American 259: 58–65. 10.1038/scientificamerican0888-76 3064296

[35] Mumford D (1996) Pattern theory. In: Knill DC, Richards W, editors. Perception as Bayesian Inference: Cambridge University Press.

[36] Leclerc YG (1989) Constructing simple stable descriptions for image partitioning. International Journal of Computer Vision 3: 73–102.

[37] Adelson EH (2000) Lightness perception and lightness illusions. In: Gazzaniga M, editor. The new cognitive neurosciences, 2nd ed. Cambridge, MA: MIT Press. pp. 339–351.

[38] Beck J, Prazdny S (1981) Highlights and the perception of glossiness. Perception & Psychophysics 30: 407–410. 7322822 10.3758/bf03206160

[39] Todd J, Norman J, Mingolla E (2004) Lightness constancy in the presence of specular highlights. Psychological Science 15: 33–39. 14717829 10.1111/j.0963-7214.2004.01501006.x

[40] Marlow P, Kim J, Anderson B (2011) The role of brightness and orientation congruence in the perception of surface gloss. Journal of Vision 11: 16.1–12 10.1167/11.9.16 21873616

[41] Anderson BL, Kim J (2009) Image statistics do not explain the perception of gloss and lightness. Journal of vision 9: 10.11–17. 10.1167/9.11.10 20053073

[42] Kim J, Marlow P, Anderson BL (2011) The perception of gloss depends on highlight congruence with surface shading. Journal of Vision 11: 4.1–19 10.1167/11.9.4 21841140

[43] O'Shea JP, Agrawala M, Banks MS (2010) The influence of shape cues on the perception of lighting direction. Journal of Vision 10: 21.1–21 10.1167/10.12.21 PMC3066057 21047753

[44] Ostrovsky Y, Cavanagh P, Sinha P (2005) Perceiving illumination inconsistencies in scenes. Perception 34: 1301–1314. 16358419 10.1068/p5418

[45] (1996) Perception as Bayesian Inference: Cambridge University Press.

[46] Ramachandran VS (1985) The neurobiology of perception. Perception 14: 97–103. 4069953 10.1068/p140097

[47] Adams WJ, Mamassian P (2004) Bayesian combination of ambiguous shape cues. Journal of Vision 4: 921–929. 15595895 10.1167/4.10.7

[48] Champion RA, Adams WJ (2007) Modification of the convexity prior but not the light-from-above prior in visual search with shaded objects. Journal of Vision 7: 10.1–10 10.1167/7.13.10 17997638

[49] Langer MS, Bulthoff HH (2001) A prior for global convexity in local shape-from-shading. Perception 30: 403–410. 11383189 10.1068/p3178

[50] Thomas R, Nardini M, Mareschal D (2010) Interactions between “light-from-above” and convexity priors in visual development. Journal of Vision 10: 6.1–7 10.1167/10.8.6 20884581

[51] Adams W, Kerrigan I, Graf E (2010) Efficient Visual Recalibration from Either Visual or Haptic Feedback: The Importance of Being Wrong. Journal of Neuroscience 30: 14745–14749. 21048133 10.1523/JNEUROSCI.2749-10.2010 PMC6633618

[52] Sun J, Perona P (1998) Where is the sun? Nature Neuroscience 1: 183–184. 10195141 10.1038/630

[53] Kerrigan IS, Adams WJ (2013) Learning different light prior distributions for different contexts. Cognition 127: 99–104. 23376295 10.1016/j.cognition.2012.12.011

