# Supplementary material for: Effects of Specular Highlights on Perceived Surface Convexity
Source: PLoS Comput Biol. 2014 May 8;10(5):e1003576. doi: 10.1371/journal.pcbi.1003576 (PMC4014396; doi:10.1371/journal.pcbi.1003576)
Supplement: Text S1 — Model equations: The equations for the full model. (PDF) [file pcbi.1003576.s003.pdf]

Supporting Information: Equations for the model

We summarize the variables in the model below. Since there are two objects in our stimuli, there may be up to two instances of each variable – these are individuated by a subscript, e.g., δ_1, δ_2. To make the notation a bit more concise, we code the binary variables with values 1 or 2 as indicated.

Observable Variables

Binary Variables:

		1	2
δ	Presence of a highlight	Absent	Present

Orientation Variables:

θ	Orientation of the shading gradient
γ	Orientation of the highlight

Hidden Variables

Binary Variables

		1	2
τ	Sign of surface curvature	Convex	Concave
χ	Specular index of surface	Matte	Glossy
α	Number of main illuminants	1	2
β	Presence of a local illuminant	Absent	Present

Orientation Variables

ϕ	Illuminant tilt

The prior over the illuminant tilt ϕ is modelled by a von Mises distribution with mean μ and concentration κ_ϕ.

Let $p(\theta \mid \tau, \phi)$ and $p(\gamma \mid \chi = 2, \phi)$ model the angular noise in how the light source produces gradients and highlights. We model this noise as a 0-mean von Mises distribution with concentration parameter κ_n.

When the parameters of the model are fixed, the probability of concave/convex response can be determined by computing the normalized product of the likelihood of all observed variables conditioned on $\tau = 1$ and $\tau = 2$, respectively, and the prior over τ, while marginalizing over all other hidden variables. In particular, letting Ξ and Ψ represent the set of observable and nuisance hidden variables, respectively, we have:

$$p(\tau \mid \Xi) \propto p(\tau) p(\Xi \mid \tau),$$

where

$$p(\Xi \mid \tau) = \int p(\Xi \mid \tau, \Psi) p(\Psi) d\Psi.$$

The likelihoods $p(\Xi \mid \tau)$ involve integrals over products of von Mises distributions. As an example, consider

$$
\begin{aligned}
& p(\theta,\gamma \mid \tau=1,\chi=2) \\
& \propto \int p(\theta,\gamma \mid \tau=1,\chi=2,\phi)p(\phi)d\phi \\
& = \int p(\theta \mid \tau=1,\phi)p(\gamma \mid \tau=1,\chi=2,\phi)p(\phi)d\phi \\
& = \frac{1}{(2\pi)^3 I_0(\kappa_n)^2 I_0(\kappa_\phi)} \int \exp\left(\kappa_n\left(\cos(\theta-\phi)+\cos(\gamma-\phi)\right)+\kappa_\phi\cos(\phi-\phi_0)\right)d\phi \\
& = \frac{1}{(2\pi)^3 I_0(\kappa_n)^2 I_0(\kappa_\phi)} \int \exp\left(\kappa_p\cos(\phi-\phi_p)\right)d\phi \\
& = \frac{I_0(\kappa_p)}{(2\pi)^2 I_0(\kappa_n)^2 I_0(\kappa_\phi)}
\end{aligned}
$$

where

$$
\begin{aligned}
\kappa_p^2 &= x_p^2 + y_p^2 \\
x_p &= \kappa_n(\cos\theta+\cos\gamma)+\kappa_\phi\cos\phi_0 \\
y_p &= \kappa_n(\sin\theta+\sin\gamma)+\kappa_\phi\sin\phi_0
\end{aligned}
$$

and I_0 is the modified Bessel function of order 0.

This type of integral emerges for each possible configuration of the hidden variables. Computing the probability of convex/concave response thus involves:

1. Solving an integral of this general form to determine a likelihood for the observable orientation variables for each possible configuration of the hidden binary variables.
2. For concave surface configurations, multiplying each of these likelihoods by a factor involving p_{os} and/or p_{om} that accounts for the likelihood of the observed (or non-observed) highlights.
3. Multiplying each joint likelihood by the priors for each of the hidden binary variables in the configuration.
4. Summing all terms where $\tau = 1$ to compute the relative probability of convex response.
5. Summing all terms where $\tau = 2$ to compute the relative probability of concave response.
6. Normalizing these two probabilities to sum to 1.

We divide the possible scenarios into four main cases, corresponding to the four conditions of Experiment 1:

1. MM: Neither object has a highlight.
2. SM: The target object has a highlight, the distractor object does not.
3. MS: The target object does not have a highlight, the distractor object does.
4. SS: Both objects have highlights.

Each of these cases is divided into subcases depending upon the values assumed by the hidden binary variables.

Case 1 (MM). $\delta_1 = \delta_2 = 1$

Here we have

$$p(\theta_1, \theta_2, \delta_1, \delta_2 | \tau_1, \tau_2, \alpha, \beta_1, \chi_1, \beta_2, \chi_2) = p(\delta_1 | \tau_1, \beta_1, \chi_1) p(\delta_2 | \tau_2, \beta_2, \chi_2) p(\theta_1, \theta_2 | \tau_1, \tau_2, \alpha)$$

where
$p(\delta_i = 1 \,|\tau_i, \beta_i = 1, \chi_i = 1) = 1$
$p(\delta_i = 1 \,|\tau_i = 2, \beta_i = 1, \chi_i = 2) = p_{os}$
$p(\delta_i = 1 \,|\tau_i = 2, \beta_i = 2, \chi_i = 1) = p_{om}$
$p(\delta_i = 1 \,|\tau_i = 2, \beta_i = 2, \chi_i = 2) = p_{os} p_{om}$
and $p(\delta_i = 1) = 0$ for all other configurations.

It remains to determine the likelihood for the shading gradient $p(\theta_1, \theta_2 | \tau_1, \tau_2, \alpha)$.

Case 1.1 $\alpha = 1$ (one main illuminant)

$$p(\theta_1, \theta_2 \,|\, \tau_1, \tau_2, \alpha = 1) = \frac{I_0(\kappa_p)}{(2\pi)^2 I_0(\kappa_n)^2 I_0(\kappa_\phi)}$$

where

$$\kappa_p^2 = x_p^2 + y_p^2$$
$$x_p = \kappa_n (\cos\theta_1' + \cos\theta_2') + \kappa_\phi \cos\phi_0$$
$$y_p = \kappa_n (\sin\theta_1' + \sin\theta_2') + \kappa_\phi \sin\phi_0$$

and

$$\theta_i' = \theta_i \text{ if } \tau_i = 1$$
$$= \theta_i + \pi \text{ if } \tau_i = 2$$

Case 1.2 $\alpha = 2$ (two windowed illuminants)

$$p(\theta_1, \theta_2 \,|\, \tau_1, \tau_2, \alpha = 2) = \frac{I_0(\kappa_{p1}) I_0(\kappa_{p2})}{(2\pi)^2 I_0(\kappa_n)^2 I_0(\kappa_\phi)^2}$$

where

$$\kappa_{pi}^2 = x_{pi}^2 + y_{pi}^2$$
$$x_{pi} = \kappa_n \cos\theta_i' + \kappa_\phi \cos\phi_0$$
$$y_{pi} = \kappa_n \sin\theta_i' + \kappa_\phi \sin\phi_0$$

Case 2 (SM). $\delta_1 = 2, \delta_2 = 1$

Here we have

$$p(\theta_1, \theta_2, \delta_1, \delta_2, \gamma_1 | \tau_1, \tau_2, \alpha, \beta_1, \chi_1, \beta_2, \chi_2) = p(\theta_1, \theta_2, \delta_1, \gamma_1 | \tau_1, \tau_2, \alpha, \beta_1, \chi_1) p(\delta_2 | \tau_2, \beta_2, \chi_2)$$

where $p(\delta_2 | \tau_2, \beta_2, \chi_2)$ is given by the equations listed for Case 1. It remains to determine $p(\theta_1, \theta_2, \delta_1, \gamma_1 | \tau_1, \tau_2, \alpha, \beta_1, \chi_1)$.

Case 2.1 $\alpha = 1$.

Case 2.1.1 $\beta_1=1, \chi_1=2$ (no local illuminant, object is shiny).

$$p(\theta_1, \theta_2, \delta_1, \gamma_1 | \tau_1 = 1, \tau_2, \alpha = 1, \beta_1 = 1, \chi_1 = 2) = \frac{I_0(\kappa_p)}{(2\pi)^3 I_0(\kappa_n)^3 I_0(\kappa_\phi)}$$

$$p(\theta_1, \theta_2, \delta_1, \gamma_1 | \tau_1 = 2, \tau_2, \alpha = 1, \beta_1 = 1, \chi_1 = 2) = (1 - p_{os}) \frac{I_0(\kappa_p)}{(2\pi)^3 I_0(\kappa_n)^3 I_0(\kappa_\phi)}$$

where

$$\kappa_p^2 = x_p^2 + y_p^2$$
$$x_p = \kappa_n (\cos\theta_1' + \cos\theta_2' + \cos\gamma_1') + \kappa_\phi \cos\phi_0$$
$$y_p = \kappa_n (\sin\theta_1' + \sin\theta_2' + \sin\gamma_1') + \kappa_\phi \sin\phi_0$$

and

$$\gamma_i' = \gamma_i \text{ if } \tau_i = 1$$
$$= \gamma_i + \pi \text{ if } \tau_i = 2$$

Case 2.1.2 $\beta_1=2, \chi_1=1$ (local illuminant, object is matte)

$$p(\theta_1, \theta_2, \delta_1, \gamma_1 | \tau_1 = 1, \tau_2, \alpha = 1, \beta_1 = 2, \chi_1 = 1) = \frac{I_0(\kappa_p)}{(2\pi)^3 I_0(\kappa_n)^2 I_0(\kappa_\phi)}$$

$$p(\theta_1, \theta_2, \delta_1, \gamma_1 | \tau_1 = 2, \tau_2, \alpha = 1, \beta_1 = 2, \chi_1 = 1) = (1 - p_{om}) \frac{I_0(\kappa_p)}{(2\pi)^3 I_0(\kappa_n)^2 I_0(\kappa_\phi)}$$

where

$$\kappa_p^2 = x_p^2 + y_p^2$$
$$x_p = \kappa_n (\cos\theta_1' + \cos\theta_2') + \kappa_\phi \cos\phi_0$$
$$y_p = \kappa_n (\sin\theta_1' + \sin\theta_2') + \kappa_\phi \sin\phi_0$$

Case 2.1.3 $\beta_1=2, \chi_1=2$ (local illuminant, object is shiny)

$$p(\theta_1, \theta_2, \delta_1, \gamma_1 | \tau_1 = 1, \tau_2, \alpha = 1, \beta_1 = 2, \chi_1 = 2) = 0$$

$$p(\theta_1, \theta_2, \delta_1 = 2, \gamma_1 | \tau_1 = 2, \tau_2, \alpha = 1, \beta_1 = 2, \chi_1 = 2)$$

$$= p_{om} p(\theta_1,\theta_2,\delta_1 = 2,\gamma_1 \mid \tau_1 = 2,\tau_2,\alpha = 1,\beta_1 = 1,\chi_1 = 2)$$
$$+ p_{os} p(\theta_1,\theta_2,\delta_1 = 2,\gamma_1 \mid \tau_1 = 2,\tau_2,\alpha = 1,\beta_1 = 2,\chi_1 = 1)$$

Case 2.2 $\alpha = 2$.

Case 2.2.1 $\beta_1=1, \chi_1=2$

$$p\left(\theta_1,\theta_2,\delta_1,\gamma_1 \mid \tau_1 = 1,\tau_2,\alpha = 2,\beta_1 = 1,\chi_1 = 2\right) = \frac{I_0\left(\kappa_{p1}\right)I_0\left(\kappa_{p2}\right)}{\left(2\pi\right)^3 I_0\left(\kappa_n\right)^3 I_0\left(\kappa_\phi\right)^2}$$

$$p\left(\theta_1,\theta_2,\delta_1,\gamma_1 \mid \tau_1 = 2,\tau_2,\alpha = 2,\beta_1 = 1,\chi_1 = 2\right) = \left(1-p_{os}\right)\frac{I_0\left(\kappa_{p1}\right)I_0\left(\kappa_{p2}\right)}{\left(2\pi\right)^3 I_0\left(\kappa_n\right)^3 I_0\left(\kappa_\phi\right)^2}$$

where

$$\kappa_{pi}^2 = x_{pi}^2 + y_{pi}^2$$

where

$$x_{p1} = \kappa_n\left(\cos\theta_1' + \cos\gamma_1'\right) + \kappa_\phi \cos\theta_\phi$$
$$y_{p1} = \kappa_n\left(\sin\theta_1' + \sin\gamma_1'\right) + \kappa_\phi \sin\theta_\phi$$

and

$$x_{p2} = \kappa_n \cos\theta_2' + \kappa_\phi \cos\phi_0$$
$$y_{p2} = \kappa_n \sin\theta_2' + \kappa_\phi \sin\phi_0$$

Case 2.2.2 $\beta_1=2, \chi_1=1$.

$$p\left(\theta_1,\theta_2,\delta_1,\gamma_1 \mid \tau_1 = 1,\tau_2,\alpha = 2,\beta_1 = 2,\chi_1 = 1\right) = \frac{I_0\left(\kappa_{p1}\right)I_0\left(\kappa_{p2}\right)}{\left(2\pi\right)^3 I_0\left(\kappa_n\right)^2 I_0\left(\kappa_\phi\right)^2}$$

$$p\left(\theta_1,\theta_2,\delta_1,\gamma_1 \mid \tau_1 = 2,\tau_2,\alpha = 2,\beta_1 = 2,\chi_1 = 1\right) = \left(1-p_{om}\right)\frac{I_0\left(\kappa_{p1}\right)I_0\left(\kappa_{p2}\right)}{\left(2\pi\right)^3 I_0\left(\kappa_n\right)^2 I_0\left(\kappa_\phi\right)^2}$$

where

$$\kappa_{pi}^2 = x_{pi}^2 + y_{pi}^2$$
$$x_{pi} = \kappa_n \cos\theta_i' + \kappa_\phi \cos\phi_0$$
$$y_{pi} = \kappa_n \sin\theta_i' + \kappa_\phi \sin\phi_0$$

Case 2.2.3 $\beta_1=2, \chi_1=2$.

$$p\left(\theta_1,\theta_2,\delta_1,\gamma_1 \mid \tau_1 = 1,\tau_2,\alpha = 2,\beta_1 = 2,\chi_1 = 2\right) = 0$$

$$p(\theta_1,\theta_2,\delta_1 = 2,\gamma_1 \mid \tau_1 = 2,\tau_2,\alpha = 2,\beta_1 = 2,\chi_1 = 2)$$
$$= p_{om} p(\theta_1,\theta_2,\delta_1 = 2,\gamma_1 \mid \tau_1 = 2,\tau_2,\alpha = 2,\beta_1 = 1,\chi_1 = 2)$$
$$+ p_{os} p(\theta_1,\theta_2,\delta_1 = 2,\gamma_1 \mid \tau_1 = 2,\tau_2,\alpha = 2,\beta_1 = 2,\chi_1 = 1)$$

Case 3 (MS). $\delta_1 = 1, \delta_2 = 2$

This is identical to Case 2, with indices 1 & 2 swapped.

Case 4 (SS). $\delta_1 = 2, \delta_2 = 2$

Case 4.1 $\alpha = 1$.

Case 4.1.1 χ_1=2, β_1=1, χ_2=2, β_2=1

$$p\left(\theta_1,\theta_2,\delta_1,\gamma_1,\delta_2,\gamma_2 \mid \tau_1 = 1,\tau_2 = 1,\alpha = 1,\beta_1 = 1,\chi_1 = 2,\beta_2 = 1,\chi_2 = 2\right) = \frac{I_0\left(\kappa_p\right)}{\left(2\pi\right)^4 I_0\left(\kappa_n\right)^4 I_0\left(\kappa_\phi\right)}$$

$$p\left(\theta_1,\theta_2,\delta_1,\gamma_1,\delta_2,\gamma_2 \mid \tau_1 = 2,\tau_2 = 1,\alpha = 1,\beta_1 = 1,\chi_1 = 2,\beta_2 = 1,\chi_2 = 2\right) = \left(1-p_{os}\right)\frac{I_0\left(\kappa_p\right)}{\left(2\pi\right)^4 I_0\left(\kappa_n\right)^4 I_0\left(\kappa_\phi\right)}$$

$$p\left(\theta_1,\theta_2,\delta_1,\gamma_1,\delta_2,\gamma_2 \mid \tau_1 = 1,\tau_2 = 2,\alpha = 1,\beta_1 = 1,\chi_1 = 2,\beta_2 = 1,\chi_2 = 2\right) = \left(1-p_{os}\right)\frac{I_0\left(\kappa_p\right)}{\left(2\pi\right)^4 I_0\left(\kappa_n\right)^4 I_0\left(\kappa_\phi\right)}$$

$$p\left(\theta_1,\theta_2,\delta_1,\gamma_1,\delta_2,\gamma_2 \mid \tau_1 = 2,\tau_2 = 2,\alpha = 1,\beta_1 = 1,\chi_1 = 2,\beta_2 = 1,\chi_2 = 2\right) = \left(1-p_{os}\right)^2\frac{I_0\left(\kappa_p\right)}{\left(2\pi\right)^4 I_0\left(\kappa_n\right)^4 I_0\left(\kappa_\phi\right)}$$

where

$$\kappa_p^2 = x_p^2 + y_p^2$$

$$x_p = \kappa_n\left(\cos\theta_1' + \cos\theta_2' + \cos\gamma_1' + \cos\gamma_2'\right) + \kappa_\phi\cos\phi_0$$

$$y_p = \kappa_n\left(\sin\theta_1' + \sin\theta_2' + \sin\gamma' + \sin\gamma_2'\right) + \kappa_\phi\sin\phi_0$$

where

$$\gamma_i' = \gamma_i \text{ if } \tau_i = 1$$

$$= \gamma_i + \pi \text{ if } \tau_i = 2$$

Case 4.1.2 χ_1=1, β_1=2, χ_2=1, β_2=2

$$p\left(\theta_1,\theta_2,\delta_1,\gamma_1,\delta_2,\gamma_2 \mid \tau_1 = 1,\tau_2 = 1,\alpha = 1,\beta_1 = 2,\chi_1 = 1,\beta_2 = 2,\chi_2 = 1\right) = \frac{I_0\left(\kappa_p\right)}{\left(2\pi\right)^4 I_0\left(\kappa_n\right)^2 I_0\left(\kappa_\phi\right)}$$

$$p\left(\theta_1,\theta_2,\delta_1,\gamma_1,\delta_2,\gamma_2 \mid \tau_1=2,\tau_2=1,\alpha=1,\beta_1=2,\chi_1=1,\beta_2=2,\chi_2=1\right)=\left(1-p_{om}\right)\frac{I_0\left(\kappa_p\right)}{\left(2\pi\right)^4 I_0\left(\kappa_n\right)^2 I_0\left(\kappa_\phi\right)}$$

$$p\left(\theta_1,\theta_2,\delta_1,\gamma_1,\delta_2,\gamma_2 \mid \tau_1=1,\tau_2=2,\alpha=1,\beta_1=2,\chi_1=1,\beta_2=2,\chi_2=1\right)=\left(1-p_{om}\right)\frac{I_0\left(\kappa_p\right)}{\left(2\pi\right)^4 I_0\left(\kappa_n\right)^2 I_0\left(\kappa_\phi\right)}$$

$$p\left(\theta_1,\theta_2,\delta_1,\gamma_1,\delta_2,\gamma_2 \mid \tau_1=2,\tau_2=2,\alpha=1,\beta_1=2,\chi_1=1,\beta_2=2,\chi_2=1\right)=\left(1-p_{om}\right)^2\frac{I_0\left(\kappa_p\right)}{\left(2\pi\right)^4 I_0\left(\kappa_n\right)^2 I_0\left(\kappa_\phi\right)}$$

where

$$\kappa_p^2 = x_p^2 + y_p^2$$
$$x_p = \kappa_n\left(\cos\theta_1' + \cos\theta_2'\right) + \kappa_\phi \cos\phi_0$$
$$y_p = \kappa_n\left(\sin\theta_1' + \sin\theta_2'\right) + \kappa_\phi \sin\phi_0$$

Case 4.1.3 $\chi_1=2$, $\beta_1=1$, $\chi_2=1$, $\beta_2=2$

$$p\left(\theta_1,\theta_2,\delta_1,\gamma_1,\delta_2,\gamma_2 \mid \tau_1=1,\tau_2=1,\alpha=1,\beta_1=1,\chi_1=2,\beta_2=2,\chi_2=1\right)=\frac{I_0\left(\kappa_p\right)}{\left(2\pi\right)^4 I_0\left(\kappa_n\right)^3 I_0\left(\kappa_\phi\right)}$$

$$p\left(\theta_1,\theta_2,\delta_1,\gamma_1,\delta_2,\gamma_2 \mid \tau_1=2,\tau_2=1,\alpha=1,\beta_1=1,\chi_1=2,\beta_2=2,\chi_2=1\right)=\left(1-p_{os}\right)\frac{I_0\left(\kappa_p\right)}{\left(2\pi\right)^4 I_0\left(\kappa_n\right)^3 I_0\left(\kappa_\phi\right)}$$

$$p\left(\theta_1,\theta_2,\delta_1,\gamma_1,\delta_2,\gamma_2 \mid \tau_1=1,\tau_2=2,\alpha=1,\beta_1=1,\chi_1=2,\beta_2=2,\chi_2=1\right)=\left(1-p_{om}\right)\frac{I_0\left(\kappa_p\right)}{\left(2\pi\right)^4 I_0\left(\kappa_n\right)^3 I_0\left(\kappa_\phi\right)}$$

$$p\left(\theta_1,\theta_2,\delta_1,\gamma_1,\delta_2,\gamma_2 \mid \tau_1=2,\tau_2=2,\alpha=1,\beta_1=1,\chi_1=2,\beta_2=2,\chi_2=1\right)$$
$$=\left(1-p_{os}\right)\left(1-p_{om}\right)\frac{I_0\left(\kappa_p\right)}{\left(2\pi\right)^4 I_0\left(\kappa_n\right)^3 I_0\left(\kappa_\phi\right)}$$

where

$$\kappa_p^2 = x_p^2 + y_p^2$$
$$x_p = \kappa_n\left(\cos\theta_1' + \cos\theta_2' + \cos\gamma_1'\right) + \kappa_\phi \cos\phi_0$$
$$y_p = \kappa_n\left(\sin\theta_1' + \sin\theta_2' + \sin\gamma_1'\right) + \kappa_\phi \sin\phi_0$$

Case 4.1.4 $\chi_1=1$, $\beta_1=2$, $\chi_2=2$, $\beta_2=1$

Same as 4.1.3 except

$$\kappa_p^2 = x_p^2 + y_p^2$$

$$x_p = \kappa_n\left(\cos\theta_1' + \cos\theta_2' + \cos\gamma_2'\right) + \kappa_\phi \cos\phi_0$$

$$y_p = \kappa_n\left(\sin\theta_1' + \sin\theta_2' + \sin\gamma_2'\right) + \kappa_\phi \sin\phi_0$$

and the four subcases are multiplied by 1, $\left(1-p_{om}\right)$, $\left(1-p_{os}\right)$, and $\left(1-p_{os}\right)\left(1-p_{om}\right)$, respectively.

Case 4.1.5 $\chi_1=2$, $\beta_1=2$, ($\beta_2=1$, $\chi_2=2$) or ($\beta_2=2$, $\chi_2=1$)

$$p\left(\theta_1,\theta_2,\delta_1,\gamma_1,\delta_2,\gamma_2 \mid \tau_1=1,\tau_2,\alpha=1,\beta_1=2,\chi_1=2,\beta_2,\chi_2\right)=0$$

$$\begin{aligned}
&p(\theta_1,\theta_2,\delta_1=2,\gamma_1,\delta_2=2,\gamma_2 \mid \tau_1=2,\tau_2,\alpha=1,\beta_1=2,\chi_1=2,\beta_2,\chi_2)\\
&=p_{om}p(\theta_1,\theta_2,\delta_1=2,\gamma_1,\delta_2=2,\gamma_2 \mid \tau_1=2,\tau_2,\alpha=1,\beta_1=1,\chi_1=2,\beta_2,\chi_2)\\
&+p_{os}p(\theta_1,\theta_2,\delta_1=2,\gamma_1,\delta_2=2,\gamma_2 \mid \tau_1=2,\tau_2,\alpha=1,\beta_1=2,\chi_1=1,\beta_2,\chi_2)
\end{aligned}$$

Case 4.1.6 $\chi_2=2$, $\beta_2=2$, ($\beta_1=1$, $\chi_1=2$) or ($\beta_1=2$, $\chi_1=1$)

$$p\left(\theta_1,\theta_2,\delta_1,\gamma_1,\delta_2,\gamma_2 \mid \tau_1,\tau_2=1,\alpha=1,\beta_1,\chi_1,\beta_2=2,\chi_2=2\right)=0$$

$$\begin{aligned}
&p(\theta_1,\theta_2,\delta_1=2,\gamma_1,\delta_2=2,\gamma_2 \mid \tau_1,\tau_2=2,\alpha=1,\beta_1,\chi_1,\beta_2=2,\chi_2=2)\\
&=p_{om}p(\theta_1,\theta_2,\delta_1=2,\gamma_1,\delta_2=2,\gamma_2 \mid \tau_1,\tau_2=2,\alpha=1,\beta_1,\chi_1,\beta_2=1,\chi_2=2)\\
&+p_{os}p(\theta_1,\theta_2,\delta_1=2,\gamma_1,\delta_2=2,\gamma_2 \mid \tau_1,\tau_2=2,\alpha=1,\beta_1,\chi_1,\beta_2=2,\chi_2=1)
\end{aligned}$$

Case 4.1.7 $\chi_1=2$, $\beta_1=2$, $\chi_2=2$, $\beta_2=2$

$$p\left(\theta_1,\theta_2,\delta_1,\gamma_1,\delta_2,\gamma_2 \mid \tau_1=1,\tau_2,\alpha=1,\beta_1=2,\chi=2_1,\beta_2=2,\chi_2=2\right)=0$$

$$p\left(\theta_1,\theta_2,\delta_1,\gamma_1,\delta_2,\gamma_2 \mid \tau_1,\tau_2=1,\alpha=1,\beta_1=2,\chi=2_1,\beta_2=2,\chi_2=2\right)=0$$

$$\begin{aligned}
&p(\theta_1,\theta_2,\delta_1=2,\gamma_1,\delta_2=2,\gamma_2 \mid \tau_1=2,\tau_2=2,\alpha=1,\beta_1=2,\chi_1=2,\beta_2=2,\chi_2=2)\\
&=p_{om}^2p(\theta_1,\theta_2,\delta_1=2,\gamma_1,\delta_2=2,\gamma_2 \mid \tau_1=2,\tau_2=2,\alpha=1,\beta_1=1,\chi_1=2,\beta_2=1,\chi_2=2)\\
&+p_{os}^2p(\theta_1,\theta_2,\delta_1=2,\gamma_1,\delta_2=2,\gamma_2 \mid \tau_1=2,\tau_2=2,\alpha=1,\beta_1=2,\chi_1=1,\beta_2=2,\chi_2=1)\\
&+p_{os}p_{om}p(\theta_1,\theta_2,\delta_1=2,\gamma_1,\delta_2=2,\gamma_2 \mid \tau_1=2,\tau_2=2,\alpha=1,\beta_1=1,\chi_1=2,\beta_2=2,\chi_2=1)\\
&+p_{os}p_{om}p(\theta_1,\theta_2,\delta_1=2,\gamma_1,\delta_2=2,\gamma_2 \mid \tau_1=2,\tau_2=2,\alpha=1,\beta_1=2,\chi_1=1,\beta_2=1,\chi_2=2)
\end{aligned}$$

Case 4.2 $\alpha=2$.

Case 4.2.1 χ_1=2, β_1=1, χ_2=2, β_2=1

$$p\left(\theta_1,\theta_2,\delta_1,\gamma_1,\delta_2,\gamma_2 \mid \tau_1=1,\tau_2=1,\alpha=2,\beta_1=1,\chi_1=2,\beta_2=1,\chi_2=2\right)=\frac{I_0\left(\kappa_{p1}\right)I_0\left(\kappa_{p2}\right)}{\left(2\pi\right)^4 I_0\left(\kappa_n\right)^4 I_0\left(\kappa_\phi\right)^2}$$

$$p\left(\theta_1,\theta_2,\delta_1,\gamma_1,\delta_2,\gamma_2 \mid \tau_1=2,\tau_2=1,\alpha=2,\beta_1=1,\chi_1=2,\beta_2=1,\chi_2=2\right)=\left(1-p_{os}\right)\frac{I_0\left(\kappa_{p1}\right)I_0\left(\kappa_{p2}\right)}{\left(2\pi\right)^4 I_0\left(\kappa_n\right)^4 I_0\left(\kappa_\phi\right)^2}$$

$$p\left(\theta_1,\theta_2,\delta_1,\gamma_1,\delta_2,\gamma_2 \mid \tau_1=1,\tau_2=2,\alpha=2,\beta_1=1,\chi_1=2,\beta_2=1,\chi_2=2\right)=\left(1-p_{os}\right)\frac{I_0\left(\kappa_{p1}\right)I_0\left(\kappa_{p2}\right)}{\left(2\pi\right)^4 I_0\left(\kappa_n\right)^4 I_0\left(\kappa_\phi\right)^2}$$

$$p\left(\theta_1,\theta_2,\delta_1,\gamma_1,\delta_2,\gamma_2 \mid \tau_1=2,\tau_2=2,\alpha=2,\beta_1=1,\chi_1=2,\beta_2=1,\chi_2=2\right)=\left(1-p_{os}\right)^2\frac{I_0\left(\kappa_{p1}\right)I_0\left(\kappa_{p2}\right)}{\left(2\pi\right)^4 I_0\left(\kappa_n\right)^4 I_0\left(\kappa_\phi\right)^2}$$

where

$$\kappa_{pi}^2 = x_{pi}^2 + y_{pi}^2$$

$$x_{pi} = \kappa_n\left(\cos\theta_i' + \cos\gamma_i'\right) + \kappa_\phi \cos\phi_0$$

$$y_{pi} = \kappa_n\left(\sin\theta_i' + \sin\gamma_i'\right) + \kappa_\phi \sin\phi_0$$

Case 4.2.2 χ_1=1, β_1=2, χ_2=1, β_2=2

$$p\left(\theta_1,\theta_2,\delta_1,\gamma_1,\delta_2,\gamma_2 \mid \tau_1=1,\tau_2=1,\alpha=1,\beta_1=2,\chi_1=1,\beta_2=2,\chi_2=1\right)=\frac{I_0\left(\kappa_{p1}\right)I_0\left(\kappa_{p2}\right)}{\left(2\pi\right)^4 I_0\left(\kappa_n\right)^2 I_0\left(\kappa_\phi\right)^2}$$

$$p\left(\theta_1,\theta_2,\delta_1,\gamma_1,\delta_2,\gamma_2 \mid \tau_1=2,\tau_2=1,\alpha=2,\beta_1=2,\chi_1=1,\beta_2=2,\chi_2=1\right)=\left(1-p_{om}\right)\frac{I_0\left(\kappa_{p1}\right)I_0\left(\kappa_{p2}\right)}{\left(2\pi\right)^4 I_0\left(\kappa_n\right)^2 I_0\left(\kappa_\phi\right)^2}$$

$$p\left(\theta_1,\theta_2,\delta_1,\gamma_1,\delta_2,\gamma_2 \mid \tau_1=1,\tau_2=2,\alpha=2,\beta_1=2,\chi_1=1,\beta_2=2,\chi_2=1\right)=\left(1-p_{om}\right)\frac{I_0\left(\kappa_{p1}\right)I_0\left(\kappa_{p2}\right)}{\left(2\pi\right)^4 I_0\left(\kappa_n\right)^2 I_0\left(\kappa_\phi\right)^2}$$

$$p\left(\theta_1,\theta_2,\delta_1,\gamma_1,\delta_2,\gamma_2 \mid \tau_1=2,\tau_2=2,\alpha=2,\beta_1=2,\chi_1=1,\beta_2=2,\chi_2=1\right)=\left(1-p_{om}\right)^2\frac{I_0\left(\kappa_{p1}\right)I_0\left(\kappa_{p2}\right)}{\left(2\pi\right)^4 I_0\left(\kappa_n\right)^2 I_0\left(\kappa_\phi\right)^2}$$

where

$$\kappa_{pi}^2=x_{pi}^2+y_{pi}^2$$
$$x_{pi}=\kappa_n\cos\theta_i'+\kappa_\phi\cos\phi_0$$
$$y_{pi}=\kappa_n\sin\theta_i'+\kappa_\phi\sin\phi_0$$

Case 4.2.3 $\chi_1=2,\ \beta_1=1,\ \chi_2=1,\ \beta_2=2$

$$p\left(\theta_1,\theta_2,\delta_1,\gamma_1,\delta_2,\gamma_2 \mid \tau_1=1,\tau_2=1,\alpha=2,\beta_1=1,\chi_1=2,\beta_2=2,\chi_2=1\right)=\frac{I_0\left(\kappa_{p1}\right)I_0\left(\kappa_{p2}\right)}{\left(2\pi\right)^4 I_0\left(\kappa_n\right)^3 I_0\left(\kappa_\phi\right)^2}$$

$$p\left(\theta_1,\theta_2,\delta_1,\gamma_1,\delta_2,\gamma_2 \mid \tau_1=2,\tau_2=1,\alpha=2,\beta_1=1,\chi_1=2,\beta_2=2,\chi_2=1\right)=\left(1-p_{os}\right)\frac{I_0\left(\kappa_{p1}\right)I_0\left(\kappa_{p2}\right)}{\left(2\pi\right)^4 I_0\left(\kappa_n\right)^3 I_0\left(\kappa_\phi\right)^2}$$

$$p\left(\theta_1,\theta_2,\delta_1,\gamma_1,\delta_2,\gamma_2 \mid \tau_1=1,\tau_2=2,\alpha=2,\beta_1=1,\chi_1=2,\beta_2=2,\chi_2=1\right)=\left(1-p_{om}\right)\frac{I_0\left(\kappa_{p1}\right)I_0\left(\kappa_{p2}\right)}{\left(2\pi\right)^4 I_0\left(\kappa_n\right)^3 I_0\left(\kappa_\phi\right)^2}$$

$$p\left(\theta_1,\theta_2,\delta_1,\gamma_1,\delta_2,\gamma_2 \mid \tau_1=2,\tau_2=2,\alpha=2,\beta_1=1,\chi_1=2,\beta_2=2,\chi_2=1\right)$$
$$=\left(1-p_{os}\right)\left(1-p_{om}\right)\frac{I_0\left(\kappa_{p1}\right)I_0\left(\kappa_{p2}\right)}{\left(2\pi\right)^4 I_0\left(\kappa_n\right)^3 I_0\left(\kappa_\phi\right)^2}$$

where

$$\kappa_{pi}^2=x_{pi}^2+y_{pi}^2$$
$$x_{p1}=\kappa_n\left(\cos\theta_1'+\cos\gamma_1'\right)+\kappa_\phi\cos\phi_0$$
$$y_{p1}=\kappa_n\left(\sin\theta_1'+\sin\gamma_1'\right)+\kappa_\phi\sin\phi_0$$

and

$$x_{p2}=\kappa_n\cos\theta_2'+\kappa_\phi\cos\phi_0$$
$$y_{p2}=\kappa_n\sin\theta_2'+\kappa_\phi\sin\phi_0$$

Case 4.2.4 $\beta_1=2,\chi_1=1,\beta_1=1,\chi_1=2$

Same as 4.2.3 except

$$\kappa_{pi}^2 = x_{pi}^2 + y_{pi}^2$$
$$x_{p2} = \kappa_n \left(\cos\theta_2' + \cos\gamma_2'\right) + \kappa_\phi \cos\phi_0$$
$$y_{p2} = \kappa_n \left(\sin\theta_2' + \sin\gamma_2'\right) + \kappa_\phi \sin\phi_0$$
and
$$x_{p1} = \kappa_n \cos\theta_1' + \kappa_\phi \cos\phi_0$$
$$y_{p1} = \kappa_n \sin\theta_1' + \kappa_\phi \sin\phi_0$$
and multipliers for the four cases are 1, $\left(1-p_{om}\right)$, $\left(1-p_{os}\right)$, and $\left(1-p_{os}\right)\left(1-p_{om}\right)$, respectively.

Case 4.2.5 $\chi_1=2$, $\beta_1=2$, ($\beta_2=1$, $\chi_2=2$) or ($\beta_2=2$, $\chi_2=1$)

$$p\left(\theta_1,\theta_2,\delta_1,\gamma_1,\delta_2,\gamma_2 \mid \tau_1 = 1,\tau_2,\alpha = 2,\beta_1 = 2,\chi_1 = 2,\beta_2,\chi_2\right) = 0$$
$$p(\theta_1,\theta_2,\delta_1 = 2,\gamma_1,\delta_2 = 2,\gamma_2 \mid \tau_1 = 2,\tau_2,\alpha = 2,\beta_1 = 2,\chi_1 = 2,\beta_2,\chi_2)$$
$$= p_{om}p(\theta_1,\theta_2,\delta_1 = 2,\gamma_1,\delta_2 = 2,\gamma_2 \mid \tau_1 = 2,\tau_2,\alpha = 2,\beta_1 = 1,\chi_1 = 2,\beta_2,\chi_2)$$
$$+p_{os}p(\theta_1,\theta_2,\delta_1 = 2,\gamma_1,\delta_2 = 2,\gamma_2 \mid \tau_1 = 2,\tau_2,\alpha = 2,\beta_1 = 2,\chi_1 = 1,\beta_2,\chi_2)$$

Case 4.2.6 $\chi_2=2$, $\beta_2=2$, ($\beta_1=1$, $\chi_1=2$) or ($\beta_1=2$, $\chi_1=1$)

$$p\left(\theta_1,\theta_2,\delta_1,\gamma_1,\delta_2,\gamma_2 \mid \tau_1,\tau_2 = 1,\alpha = 2,\beta_1,\chi_1,\beta_2 = 2,\chi_2 = 2\right) = 0$$
$$p(\theta_1,\theta_2,\delta_1 = 2,\gamma_1,\delta_2 = 2,\gamma_2 \mid \tau_1,\tau_2 = 2,\alpha = 2,\beta_1,\chi_1,\beta_2 = 2,\chi_2 = 2)$$
$$= p_{om}p(\theta_1,\theta_2,\delta_1 = 2,\gamma_1,\delta_2 = 2,\gamma_2 \mid \tau_1,\tau_2 = 2,\alpha = 2,\beta_1,\chi_1,\beta_2 = 1,\chi_2 = 2)$$
$$= p_{os}p(\theta_1,\theta_2,\delta_1 = 2,\gamma_1,\delta_2 = 2,\gamma_2 \mid \tau_1,\tau_2 = 2,\alpha = 2,\beta_1,\chi_1,\beta_2 = 2,\chi_2 = 1)$$

Case 4.2.7 $\chi_1=2$, $\beta_1=2$, $\chi_2=2$, $\beta_2=2$

$$p\left(\theta_1,\theta_2,\delta_1,\gamma_1,\delta_2,\gamma_2 \mid \tau_1 = 1,\tau_2,\alpha = 2,\beta_1 = 2,\chi = 2_1,\beta_2 = 2,\chi_2 = 2\right) = 0$$
$$p\left(\theta_1,\theta_2,\delta_1,\gamma_1,\delta_2,\gamma_2 \mid \tau_1,\tau_2 = 1,\alpha = 2,\beta_1 = 2,\chi = 2_1,\beta_2 = 2,\chi_2 = 2\right) = 0$$

$$p(\theta_1,\theta_2,\delta_1 = 2,\gamma_1,\delta_2 = 2,\gamma_2 \mid \tau_1 = 2,\tau_2 = 2,\alpha = 2,\beta_1 = 2,\chi_1 = 2,\beta_2 = 2,\chi_2 = 2)$$
$$= p_{om}^2 p(\theta_1,\theta_2,\delta_1 = 2,\gamma_1,\delta_2 = 2,\gamma_2 \mid \tau_1 = 2,\tau_2 = 2,\alpha = 2,\beta_1 = 1,\chi_1 = 2,\beta_2 = 1,\chi_2 = 2)$$
$$+p_{os}^2 p(\theta_1,\theta_2,\delta_1 = 2,\gamma_1,\delta_2 = 2,\gamma_2 \mid \tau_1 = 2,\tau_2 = 2,\alpha = 2,\beta_1 = 2,\chi_1 = 1,\beta_2 = 2,\chi_2 = 1)$$
$$+p_{os}p_{om}p(\theta_1,\theta_2,\delta_1 = 2,\gamma_1,\delta_2 = 2,\gamma_2 \mid \tau_1 = 2,\tau_2 = 2,\alpha = 2,\beta_1 = 1,\chi_1 = 2,\beta_2 = 2,\chi_2 = 1)$$
$$+p_{os}p_{om}p(\theta_1,\theta_2,\delta_1 = 2,\gamma_1,\delta_2 = 2,\gamma_2 \mid \tau_1 = 2,\tau_2 = 2,\alpha = 2,\beta_1 = 2,\chi_1 = 1,\beta_2 = 1,\chi_2 = 2)$$
